# Refining biomarker-based clustering of cardiovascular inflammatory phenotypes in HIV using Recursive Feature Addition: A comparative evaluation approach

**DOI:** 10.1371/journal.pcbi.1014209

**Published:** 2026-04-27

**Authors:** Rachel Mac Cann, Dana Alalwan, Gurvin Saini, Alejandro Abner Garcia Leon, Neeltje A. Kootstra, Padraig McGettrick, Aoife G Cotter, Alan Winston, Peter Reiss, Caroline Sabin, Patrick W. Mallon, on behalf of the UPBEAT-CAD, AIID and COBRA cohorts

**Affiliations:** 1 School of Medicine, University College Dublin, Belfield, Dublin, Ireland; 2 Department of Infectious Diseases, St Vincent’s University Hospital, Elm Park, Dublin, Ireland; 3 Centre for Experimental Pathogen Host Research (CEPHR), University College Dublin, Belfield, Dublin, Ireland; 4 Amsterdam Institute for Immunology and Infectious Diseases, Amsterdam, The Netherlands; 5 Department of Experimental Immunology, Amsterdam University Medical Centers, University of Amsterdam, Amsterdam, The Netherlands; 6 Department of Infectious Diseases, Mater Misericordiae University Hospital, Dublin, Ireland; 7 Department of Infectious Disease, Imperial College London, London, United Kingdom; 8 Amsterdam UMC location University of Amsterdam, Global Health, Amsterdam, The Netherlands; 9 Amsterdam Institute for Global Health and Development, Amsterdam, The Netherlands; 10 Institute for Global Health, University College London, London, United Kingdom; Xinjiang Technical Institute of Physics and Chemistry, CHINA

## Abstract

**Background:**

People living with HIV remain at elevated risk for a number of non-communicable diseases, including cardiovascular disease (CVD), driven in part by chronic inflammation. While prior studies have identified inflammatory biomarker patterns linked to CVD in people with HIV, it remains unclear which combinations of biomarkers most effectively predict clinical outcomes. We aimed to develop and evaluate a framework for refining biomarker-based clustering approaches to better capture inflammatory patterns associated with a cardiovascular phenotype (CVP) in people with HIV.

**Methods:**

We developed and evaluated three recursive feature addition (RFA) models to enhance biomarker-driven clustering of people with and without HIV. Using a 24-marker initial panel of biomarkers chosen for their links to clinical CVP in people with HIV, we compared three models for selective inclusion of 31 additional, exploratory biomarkers: (1) a stepwise additive model evaluating biomarkers cumulatively based on biological relevance; (2) a stepwise additive model evaluating biomarkers individually; and (3) a greedy forward-backward selection model. Each model was assessed using principal component analysis (PCA), cluster stability, biological coherence and association with a CVP and 10-year Atherosclerotic Cardiovascular Disease (ASCVD) risk.

**Results:**

All three RFA models generated three, biomarker-derived clusters. Post RFA cluster biomarker composition, model stability and clinical associations of these clusters differed across models. The individual additive model (Model 2) produced the most distinct separation of inflammatory profiles, incorporating 11 additional biomarkers, including, GDF-15, IFN-λ2 and Thrombopoietin). In this model, Cluster 3 was characterised by heightened innate and adaptive immune activation, the highest CVP prevalence (11%) and the strongest association with CVP (adjusted odds ratio (aOR) 2.3, 95% CI 1.04–5.09).

**Conclusion:**

We demonstrate that an RFA framework using a stepwise, additive model evaluating biomarkers individually to enhance clustering profiles provides optimal unsupervised clustering of exploratory biomarkers to reveal additional associations between inflammatory patterns and CVP in people with and without HIV.

## Author summary

People living with HIV are living longer, healthier lives thanks to effective treatment, but they remain at greater risk of developing non-communicable diseases, such as cardiovascular disease. This risk appears to be linked to ongoing systemic inflammation and previous studies have shown that people with HIV often have higher levels of circulating inflammatory biomarkers. Understanding which of these biomarkers, or combinations of them, best identify people at higher risk is an important step toward more personalised care, but is also a challenging computational problem. In our study, we used a computer-based approach called recursive feature addition to explore whether adding new biomarkers to an existing panel associated with cardiovascular disease could improve how individuals are grouped based on their inflammatory profiles. Combining data from three large international cohort studies, we compared different modelling strategies and found that one approach produced particularly distinct and biologically meaningful clusters, revealing a subgroup with higher inflammation and greater cardiovascular disease risk. Our findings demonstrate how data-driven feature selection can refine the identification of biological patterns linked to disease, bridging computational modelling with clinical understanding.

## Introduction

Worldwide, people living with HIV are living longer due to the success of antiretroviral therapy (ART) [1]. However, despite these advances, people with HIV remain at increased risk of non-communicable diseases (NCDs) such as cardiovascular disease (CVD), metabolic syndrome, certain cancers, and cognitive impairment, likely reflecting the combined effects of chronic inflammation and traditional risk factors. CVD has now emerged as a leading cause of death in people with HIV receiving ART [2], with traditional CVD risk factors, although prevalent in this group, not fully accounting for this excess risk [3].

Chronic inflammation and immune activation are hallmarks of HIV infection, persisting even in individuals on ART with fully suppressed viral replication [4,5]. Elevated markers of a number of inflammatory pathways, such as innate immune activation (sCD14) and systemic inflammation (hsCRP, IL-6), have been linked to the development of subclinical and clinical coronary artery disease (CAD) in people with HIV [6]. Although associations between single biomarkers and CVD risk have been well documented among people with HIV [7,8], discovery of a single biomarker that fully reflects the complex, multifactorial nature of inflammation has remained elusive, with limited predictive accuracy for CVD risk. A more integrated approach to understanding how multiple inflammatory pathways interact and contribute to the development of NCD in people with HIV is only beginning to emerge.

An increasing number of studies have measured multiple biomarkers to identify inflammatory patterns associated with higher CVD and NCD risk, rather than relying on single-marker analyses [5,9,10]. Previous analysis within our group have identified three distinct biomarker-derived inflammatory patterns associated with both subclinical CAD (measured by CT coronary angiography) and prevalent CVD events [10]. One cluster, characterised by elevated markers of gut epithelial barrier disruption (I-FABP), T-cell activation, and systemic inflammation, was particularly notable for its strong association with increased coronary artery plaque burden (CAP) and clinical CVD, even after adjustment for traditional CVD risk factors and HIV status [10]. These findings were validated in the Pharmacokinetic and clinical Observations in PeoPle over FiftY (POPPY) study in the UK/Ireland, which also identified a three clusters, one of which was also associated with higher estimated CVD risk [5]. An analysis of the AGEhIV cohort in the Netherlands also demonstrated that a preserved-thymic/low-inflammation cluster in people with HIV was linked to lower comorbidity burden including CVD, during longitudinal follow-up [9]. These studies suggest that a people with HIV can be stratified into higher and lower risk cardiovascular categories based on distinct inflammatory profiles, independent to conventional CVD risk factors.

However, while these prior studies highlight the relevance of key inflammatory pathways, it remains unclear which individual biomarkers, or their combinations, most robustly associate with clinical outcomes. As high-throughput platforms have introduced a new era of multiplex biomarker assessment that is feasible to implement into clinical care, it is important to determine how this expanded data can be incorporated into existing frameworks to refine disease stratification in people living with HIV.

A key challenge in this setting is that naïvely expanding biomarker panels can degrade clustering performance by introducing redundant or weakly informative features, reducing cluster stability and interpretability. Recursive feature addition (RFA) is a wrapper-based, forward feature selection strategy in which candidate features are iteratively added to an existing model and retained only if they provide incremental improvement in performance or cluster discrimination relative to a predefined baseline feature set. By evaluating the marginal contribution of each additional feature, RFA enables controlled model refinement while preserving biologically meaningful structure derived from prior knowledge. This makes RFA particularly well suited to optimising biomarker-driven clustering in high-dimensional, correlated immunological data, where the goal is improved outcome discrimination while maintaining interpretability and translational relevance.

In this study we applied a RFA strategy, comparing three different biomarker inclusion strategies to assess the contribution of new biomarkers to an existing clustering model. Our goal was to refine biomarker-based clustering by identifying additional markers that enhance the biological relevance and clinical stratification of clusters associated with cardiovascular outcomes in people with HIV.

## Methods

### Ethics statement

The AIID Cohort Study was approved by the National Research Ethics Committee in Ireland, reference 20-NREC-COV-056. The UPBEAT-CAD study was approved by the Mater Misericordiae University Hospital and Mater Private Hospital Institutional Review Board. The COBRA cohort study was approved by the institutional review board of the Academic Medical Center (AMC) (reference number NL 30802.018.09) and a UK Research Ethics Committee (REC) (reference number 13/LO/0584 Stanmore, London). Participants across all cohorts gave written informed consent.

### Dataset and study cohort

People with and without HIV from three different prospective, multicentre cohort studies were included,. This included people with HIV from the All-Ireland Infectious Diseases Cohort (AIID) study, a prospective, multicentre cohort study in Ireland. The second cohort included participants >40 years old with no known history of CVD from the Understanding the Pathology of Comorbid Disease in HIV-Infected Individuals With Coronary Artery Disease (HIV UPBEAT CAD) sub study, a cross-sectional study of people with and without HIV (that were propensity score matched for traditional cardiovascular risk factors. The third cohort included a subset of participants from the Co-morBidity in Relation to HIV/AIDS (COBRA) study, which investigated age-related comorbidities in people living with and without HIV enrolled in the AGEhIV and POPPY studies [11]. All participants with HIV were virally suppressed. All cohorts contributed blood samples, along with clinical and socioeconomic data.

Cardiovascular conditions were defined as a composite vascular phenotype (CVP), encompassing a history of hypertension, myocardial infarction, stroke, transient ischaemic attack (TIA), coronary artery disease, or peripheral vascular disease. This definition aligns with the broader vascular disease framework previously applied in the UPBEAT-CAD and AGEhIV cohort and underpinned the original biomarker selection strategy used here [10,12].To complement this composite phenotype and to capture cardiovascular risk independent of established disease, particularly given the inclusion of hypertension within this definition, the 10-year Atherosclerotic Cardiovascular Disease (ASCVD) risk score was calculated using pooled cohort equations and used as an additional outcome [13].

### Biomarker measurement and data preparation

We used quantitative immunoassays (as previously described) to measure 55 plasma biomarkers associated with specific immune and inflammatory pathways (Table 1) [10]. Biomarker data from all platforms were combined into a single dataset for integrated analysis. For markers with incomplete data (less than 10% of the overall data), missing values were imputed using multiple imputation with predictive mean matching (m = 5, 50 iterations) via the *mice* package (v3.16.0) in R [14].

**Table 1:** Full Biomarker panel.

Systemic Inflammation		T Helper 1 response	
**hsCRP**	Procalcitonin	**IL2** **IL18** **IL-12p70**	
**IL-6**	**IFN-γ**		
**IL1β**	IL-5		
IL-17	IL-7		
**TNF- α**	IL-8		
PDL-1	GM-CSF		
Innate Immune Activation		Microbial translocation	
**sCD163**	MDC	**sCD14** **LBP**	Zonulin Beta-D-Glucan
**MCP1**	CXCL9		
**MIP1a**	CXCL10		
Endothelial Activation		T cell modulation	
**ICAM-1** VEGF **E selectin**	**vWF** **sVCAM-1**	**TSLP**	
Coagulation		Gut epithelial barrier disruption	
**P selectin** **sCD40L** **D Dimer**	Thrombopoietin	**I-FABP**	
Obesity		Antiviral	
Adiponectin Leptin	Resistin FABP4	IFN-α2a IFN-β IFN-λ1	IFN-λ2 IFN-λ3
Tissue Repair		Immune Regulation	
EGF TGF- α	PDGF-AA	IL-33 IL-15	**IL1RA**
Anti-inflammatory		Neuronal Pathways	
**IL-10** GDF-15	IL-13	Beta-NGF	

CXCL, C-X-C motif chemokine ligand; EGF, Epidermal Growth Factor; E-selectin, Endothelial-Selectin; GM-CSF, Granulocyte-Macrophage Colony-Stimulating Factor; GP-6, Glycoprotein VI; GDF-15, Growth Differentiation Factor 15; hsCRP, high sensitivity C Reactive protein; IFN, Interferon; I-FABP, intestinal fatty acid binding protein; IL, Interleukin; IL1b, interleukin 1 beta; IL1RA, IL-1 receptor antagonist; LBP, LPS binding protein; MCP-1, Monocyte chemoattractant protein; MDC, Macrophage-Derived Chemokine; MIP-1, macrophage inflammatory protein; PDGF-AA, Platelet-Derived Growth Factor-AA; PD-L1, programmed death-ligand 1; P-selectin, Platelet-Selectin; RANTES, Regulated upon Activation, Normal T cell Expressed and Secreted; sCD163, soluble cluster of differentiation 163; sCD40L, soluble CD40 ligand; s-ICAM, soluble intercellular adhesion molecule; sVCAM-1, soluble Vascular Cell Adhesion Molecule-1; TNF-α, tumour necrosis factor alpha; TNFR, tumour necrosis factor receptor; TGF-α, Transforming Growth Factor-alpha; TSLP, Thymic Stromal Lymphopoietin; VCAM1, Vascular cell adhesion molecule; VEGF, Vascular Endothelial Growth Factor; vwf, von Willebrand factor.

To adjust for any plate-specific batch effects, ComBat batch correction (from the *sva* package) was applied to the entire combined dataset using an empirical Bayes framework [15]. This approach adjusts for systematic batch effects while preserving biological variation relevant to inflammatory biomarker patterns. Biomarker concentrations were log-transformed to approximate normality and scaled to unit variance to ensure that markers with larger intrinsic variability did not disproportionately influence the analysis. These preprocessing steps were performed on the full biomarker dataset prior to downstream analyses to ensure comparability of measurements across cohorts and assay batches.

### Definition of cardiovascular outcomes

Associations between biomarker-defined clusters and CVP and 10-year ASCVD risk were assessed using univariable logistic regression, with the uninflamed cluster as the reference. Multivariable models for CVP were adjusted for age, sex, smoking history (current or ex-smoker), body mass index (BMI) and dyslipidaemia (defined as elevated total cholesterol or triglycerides, or a documented history of dyslipidaemia). ASCVD risk was not adjusted for these covariates, as it is itself a composite risk score derived from established cardiovascular risk factors.

Missing data for clinical covariates (BMI and smoking history) were imputed using multiple imputation with predictive mean matching (m = 5, 50 iterations) [14]. Continuous variables were imputed using predictive mean matching, and categorical variables using classification and regression tree (CART) methods. All analyses were conducted in R version 4.3.2, and odds ratios (ORs) for the association between outcomes and biomarker cluster were reported with profile-likelihood 95% confidence intervals (95% CIs).

### Initial clustering and baseline model replication

An initial panel of 24 biomarkers was selected based on prior biomarker–based clustering analysis conducted in the UPBEAT-CAD and AIID cohorts, which identified a distinct inflammatory cluster associated with increased prevalence of both subclinical and clinical CVD (Table 1, highlighted in bold) [10]. This 24-biomarker panel was used as the common baseline input for all subsequent clustering and modelling analyses.

Clustering was performed using a consistent analytical pipeline comprising principal component analysis (PCA) for dimensionality reduction, followed by hierarchical clustering using Ward’s minimum variance method and squared Euclidean distance (11). Principal components were retained according to the default Hierarchical Clustering on Principal Components (HCPC) criterion implemented in the *FactoMineR* package, which selects a reduced set of components that capture the majority of total variance while minimising noise. In this baseline 24-biomarker model, the retained principal components collectively explained more than 70% of the total variance. The optimal number of clusters was automatically determined using the HCPC algorithm [16]. This PCA–HCPC pipeline and parameterisation were used consistently across all subsequent clustering analyses.

Following baseline clustering, biomarker contributions to cluster formation were quantified as the standardised difference between the conditional mean within a cluster and the overall mean across all participants. Biomarkers with the largest standardised differences, exceeding the threshold defined by the corresponding normal distribution quantile, were considered the most influential in defining that cluster’s profile. Within clusters, these influential biomarkers were ranked in descending order to highlight those most characteristic of the cluster-specific inflammatory pattern.

### Recursive feature addition methodology

**Fig 1:**
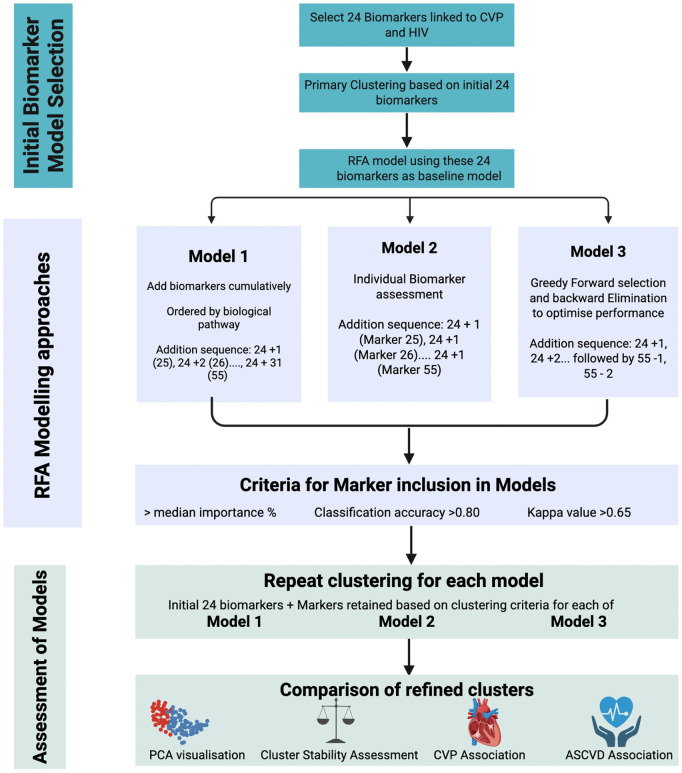
Workflow for biomarker-based clustering and recursive feature addition to characterise cardiovascular phenotypes in HIV.

To evaluate how different strategies for incorporating additional biomarkers altered cluster structure and downstream cardiovascular associations, three RFA approaches were compared (Fig 1). Each strategy extended the initial 24-biomarker model by incorporating up to 31 additional candidate biomarkers, with inclusion criteria specific to each strategy.

- Model 1. Cumulative biomarker addition guided by functional pathway relevance
- Model 2. Independent single-marker evaluation without cumulative retention
- Model 3. Iterative forward–backward selection driven by classification performance.

At each iteration, model performance was evaluated against the initial 24-biomarker model, allowing the marginal utility of each additional biomarker to be quantified.. Performance metrics, included classification accuracy and Cohen’s Kappa.

Analytical workflow used to refine biomarker-based clustering for the identification of cardiovascular phenotypes (CVP) in people with HIV. An initial set of 24 CVP-related biomarkers was selected and used for primary clustering. RFA was applied to evaluate three feature selection strategies: (i) cumulative biomarker addition guided by biological relevance, (ii) independent single-marker evaluation, and (iii) greedy forward–backward selection. Biomarkers were retained for further analysis if they met predefined performance criteria based on importance, classification accuracy, and agreement. For each model, clustering was repeated using the baseline biomarkers together with retained markers. Refined clusters were subsequently compared using principal component analysis (PCA) visualisation, cluster stability assessment, and associations with cardiovascular phenotypes and atherosclerotic cardiovascular disease (ASCVD). Created in BioRender. Alalwan, D. (2026) https://BioRender.com/ahf37oh

### Common Processing Pipeline

The primary analytical tool used for all modelling strategies was a random forest (RF) classifier [17] due to its ability to handle high-dimensional, potentially collinear predictors and its internal bootstrap-based validation framework [18]. RF models were used to evaluate the incremental contribution of candidate biomarkers to cluster classification.

Models were implemented using the *caret* framework with the *ranger* backend, with 500 trees grown per model and permutation-based variable importance enabled. For each modelling strategy, data were partitioned into training (70%) and test (30%) subsets using stratified sampling to preserve cluster proportions.

Within the training set, 3-fold cross-validation was applied to ensure internal validation and mitigate overfitting [19]. All feature selection, variable importance estimation, and model tuning steps were performed exclusively within the training data during cross-validation, while the held-out test set was used only for final model evaluation. Candidate biomarkers were retained if they demonstrated evidence of improved model performance, defined by exceeding thresholds for variable importance, classification accuracy (> 0.80), and Cohen’s kappa (> 0.65). As the biomarker data were harmonised prior to modelling, RF accuracy was interpreted as a relative measure of cluster separability rather than as an independent indicator of cluster validity. Variable importance values were normalised to obtain percentage contributions to facilitate comparison across biomarkers [20–22].

## RFA model implementation

### Model 1: Stepwise addition with cumulative evaluation

In this approach, biomarkers were added cumulatively to the initial model in a fixed sequence determined by the authors, based on their involvement in hypothesised pathways (Table 2). Beginning with the initial set of 24 biomarkers, each subsequent model iteration incorporated one additional biomarker in the predefined order (i.e., 24 + 1 (25), 24 + 2 (26),..., 24 + 31 (55)). This cumulative inclusion allowed for the assessment of incremental improvements in model performance and identification of saturation points.

**Table 2:** Sequential addition of biomarkers (25–55) used in Model 1 to the initial 24-marker model, ordered by functional relevance to biological pathways identified in initial clustering.

Order	Biomarker	Pathway
25	Beta-D-Glucan (BDG)	Microbial Translocation
26	Zonulin	Microbial Translocation
27	Thrombopoietin	Coagulation
28	VEGF	Endothelial Activation
29	MDC	Innate Immune Activation
30	CXCL9	Innate Immune Activation
31	CXCL10	Innate Immune Activation
32	IL-8	Systemic Inflammation
33	IL-17	Systemic Inflammation
34	Procalcitonin	Systemic Inflammation
35	PDL-1	Anti-inflammatory
36	IL-13	Anti-inflammatory
37	IL-5	Immune Regulation
38	IL-7	Immune Regulation
39	IL-15	Immune Regulation
40	IL-33	Immune Regulation
41	GDF-15	Immune Regulation
42	GM-CSF	Immune Regulation
43	EGF	Tissue Repair
44	TGF-α	Tissue Repair
45	PDGF-AA	Tissue Repair
46	Adiponectin	Obesity
47	Leptin	Obesity
48	Resistin	Obesity
49	FABP4	Obesity
50	IFN-α2a	Antiviral
51	IFN-β	Antiviral
52	IFN-λ1 (IL-lambda1)	Antiviral
53	IFN-λ2 (IFN-Lambda2)	Antiviral
54	IFN-λ3 (IFN-Lambda3)	Antiviral
55	Beta-NGF	Neuronal Pathways

Beta-NGF; Beta-Nerve Growth Factor, CXCL, C-X-C motif chemokine ligand; EGF, Epidermal Growth Factor; GM-CSF, FABP4; Fatty Acid-Binding Protein 4, Granulocyte-Macrophage Colony-Stimulating Factor; GDF-15, Growth Differentiation Factor 15; IFN, Interferon; IL, Interleukin; MDC, Macrophage-Derived Chemokine; PDGF-AA, Platelet-Derived Growth Factor-AA; PD-L1, programmed death-ligand 1; TGF-α, Transforming Growth Factor-alpha; VEGF, Vascular Endothelial Growth Factor;

### Model 2: Independent Addition Without Order Assumptions

This approach involved each candidate biomarker being evaluated independently by adding it to the baseline model one at a time. Unlike Model 1, no cumulative feature retention was performed; each model consisted of the 24 initial biomarkers plus one additional candidate (i.e., 24 + 1 (marker 26), 24 + 1 (marker 27),..., 24 + 1 (marker 55)).. This strategy allowed for direct comparison of the individual predictive utility of each biomarker when added in isolation to the initial core set.

### Model 3: Bidirectional feature selection

A data-driven, greedy feature selection algorithm was implemented to iteratively identify the optimal subset of biomarkers that maximised classification performance. Unlike traditional stepwise selection methods that use statistical significance (p-values) to guide inclusion or exclusion, this greedy recursive procedure relied solely on empirical model performance, specifically accuracy and Kappa, to determine which biomarkers to retain. The algorithm began with the initial 24 biomarker model and performed forward selection, sequentially testing each of the remaining 31 biomarkers one at a time. For each candidate biomarker, a new RF model was trained and its classification accuracy (and Kappa) evaluated on the held-out test set. The biomarker yielding the greatest improvement was retained and the model repeated, creating successive models of 24 + 1, 24 + 2, and so on. Once no further forward gains were achieved, a backward elimination step followed, in which previously included biomarkers were temporarily removed and the model re-evaluated. If exclusion led to improved performance, the biomarker was permanently discarded. This forward–backward iterative process continued until the model reached a performance plateau, yielding a final, parsimonious biomarker set optimised purely on empirical accuracy rather than predefined biological pathways.

### Biomarker selection stability

To assess the robustness of biomarker selection beyond a single train–test split, biomarker selection stability was evaluated for each modelling strategy. For Models 1 and 2, stability was evaluated using repeated resampling (n = 50 iterations). In each iteration, a baseline RF model using the initial 24 biomarkers was trained and evaluated on a training-only split. Candidate biomarkers (markers 25–55) were then added according to model design and re-evaluated. Selection was defined using a delta-based criterion, whereby a biomarker was considered selected in a given iteration only if it produced a minimum improvement in both classification accuracy and Cohen’s kappa relative to the baseline model (delta-based criterion). Selection frequency across resampling iterations was used as an indicator of relative stability of biomarker prioritisation within the correlated biomarker panel. As candidate biomarkers may be partially correlated, no strict a priori threshold for stable selection was imposed; instead, frequencies were interpreted comparatively across models.

For Model 3, given the path-dependent nature of greedy selection, the full forward–backward procedure was repeated across multiple resampled training–testing splits. For each iteration, the final selected biomarker set and corresponding performance were recorded. Selection frequencies were summarised to characterise the reproducibility of the greedy procedure.

### Cluster reconstruction and stability assessment

To assess the impact of model-specific biomarker sets on clustering, secondary clustering was performed separately for each RFA model. The baseline 24 biomarkers were combined with model-specific selected biomarkers, and PCA–HCPC clustering was repeated using the same pipeline and parameters as the baseline analysis. This yielded three updated clustering solutions corresponding to each model.

Cluster robustness was evaluated using bootstrap resampling (n = 500 iterations) of the biomarker matrix. For each bootstrap iteration, participants were resampled with replacement to generate datasets of equal size to the original cohort. PCA and HCPC were re-applied using the same pipeline as in the primary analysis. Cluster assignments from each bootstrap iteration were compared with those from the original solution using the Adjusted Rand Index (ARI), which quantifies agreement while correcting for chance

Iterations in which the clustering procedure failed to converge or produced invalid solutions were excluded (<2% of iterations). The resulting distribution of ARI values (median, mean, standard deviation, minimum, maximum, and interquartile range) was used to summarise cluster stability and to characterise the presence of any unstable tail in the stability distribution. Higher ARI values indicate greater agreement between bootstrap-derived and original cluster assignments, with values closer to 1 reflecting highly stable clustering solutions.

Given the multi-cohort structure of the dataset, potential cohort effects were examined using chi-squared tests for cluster–cohort association and analysis of variance to assess whether cohort explained variation along principal components.

### Cluster–outcome associations and regression stability

The clinical relevance of the updated clusters was assessed by examining associations with CVP and its components. Sensitivity analyses were conducted separately for cardiovascular disease events (myocardial infarction, stroke, TIA, CAD, or peripheral vascular disease) and for hypertension alone to account for outcome heterogeneity and differential event prevalence. Predictive validity was assessed by comparing the degree to which CVP was differentiated across clusters relative to the initial clustering solution.

To assess the robustness of downstream associations, stratified bootstrap resampling (n = 1000 iterations) was applied to adjusted logistic regression models. For each iteration, samples were resampled with replacement while preserving outcome prevalence, and models were refitted Bootstrap analyses were performed separately for CVP, cardiovascular events, and hypertension. OR distributions were summarised using medians, percentile-based confidence intervals, and the proportion of iterations in which ORs exceeded unity, providing a measure of cluster–outcome reproducibility.

## Results

### Participant characteristics

A total of 408 participants (318 (77.9%) people with HIV) were included in the analysis (Table 3). Median age was 50 (interquartile range [IQR], 43–58) years, 83% were male, 68% White, and 78% were people with HIV. Overall, 136 participants (33.3%) met criteria for a CVP, including hypertension (n = 120), coronary artery disease (n = 23), heart failure (n = 4), myocardial infarction (n = 3), peripheral artery disease (n = 3) and TIA/cerebrovascular accident (n = 2). 17 participants reported more than one type of event. Median ASCVD risk score was 6% (IQR 2.4-13.0%).

**Table 3:** Baseline demographics of combined cohorts.

Characteristic	People without HIV, N = 90^*1*^	People with HIV, N = 318^*1*^
**Age (years), median (IQR)**	55 (49, 63)	49 (40, 55)
**Sex, Male**	83 (93%)	254 (80%)
**Ethnicity**		
Asian	1 (1.1%)	12 (3.8%)
Black	4 (4.5%)	68 (22%)
Hispanic/ Brazilian	0 (0%)	44 (14%)
White	83 (94%)	192 (61%)
**CD4 Count (cells/mm** ^**3**^ **), median (IQR)**	NA	614 (481, 795)
**Nadir CD4 count (cells/mm** ^**3**^ **), median (IQR)**	NA	210 (104, 328)
**ART Duration (years), median (IQR)**	NA	10 (6, 16)
**MSM**	48 (81%)	203 (64%)
**BMI Range (kg/m** ^**2)**^		
18-24.9	36 (41%)	135 (45%)
25-29.9	34 (39%)	97 (32%)
>30	18 (20%)	71 (23%)
**Smoking**		
Current	22 (25%)	68 (22%)
Ex smoker	29 (33%)	68 (22%)
Never smoked	37 (42%)	176 (56%)
**Study Site**		
Amsterdam	46 (51%)	66 (21%)
Dublin	30 (33%)	214 (67%)
London	14 (16%)	38 (12%)
**CVP**	38 (42%)	98 (31%)
Hypertension	30 (34%)	89 (28%)
Heart failure	2 (2.3%)	2 (0.6%)
Stroke and/or transient ischemic attack	0 (0%)	4 (1.3%)
Myocardial infarction	2 (2.2%)	1 (0.3%)
Coronary artery disease	11 (12%)	12 (3.8%)
Peripheral vascular disease	0 (0%)	3 (0.9%)
**ASCVD Risk Score %, median (IQR)**	10 (4, 19)	5 (2, 12)

ASCVD, Atherosclerotic Cardiovascular Disease risk; ART, antiretroviral treatment; BMI, body mass index; CVP, cardiovascular phenotype; MSM, men having sex with men.

### Initial model evaluation

**Fig 2:**
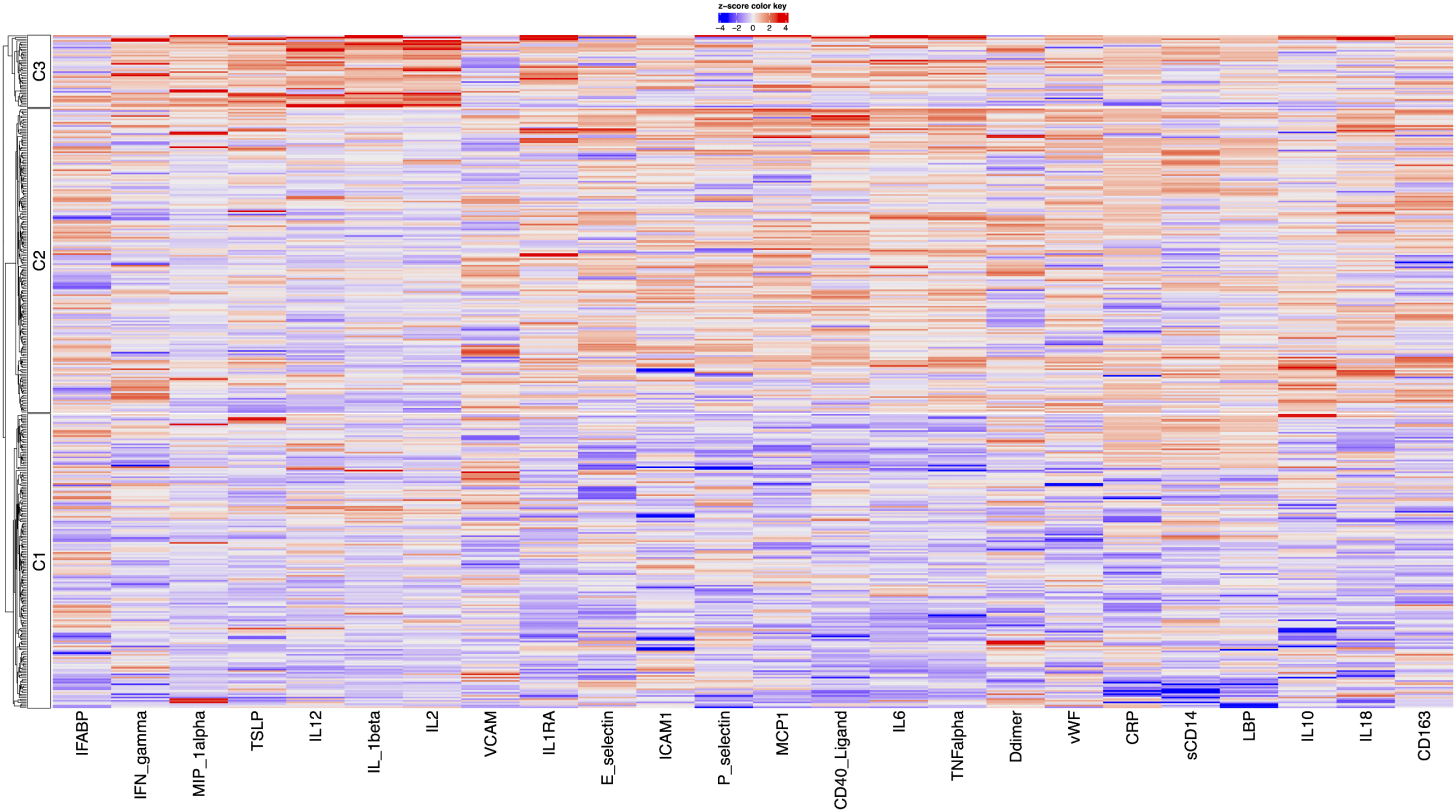
Heatmap showing biomarker contribution to cluster formation in the initial model.

Modelling the initial 24 biomarkers revealed three clusters: Cluster 1 (n = 181, 44.3%) (“Uninflamed”) exhibited low levels of inflammatory biomarkers such as soluble CD40-Ligand (sCD40L), CD163, CRP, IL18, IL6, and TNF-α (Fig 2), Cluster 2 (n = 183, 45%) showed elevated systemic inflammation and endothelial activation markers (CD40-Ligand, IL6, TNF-α, vWF, E-selectin, P-selectin) and elevated IL-10 but suppressed Th1 cytokines (IL2, IL12, IL1β) (Table 4). Cluster 3 (n = 44, 10.7%) displayed increases in both pro-inflammatory and Th1-associated pathways, including IFN-γ, IL1β, IL2, IL12, TNF-α, TSLP and MIP1α. CVP rates were comparable across clusters 2 (38%) and 3 (39%) and slightly lower in cluster 1 (28%) (p = 0.091). Median 10-year ASCVD risk scores also differed across clusters, Cluster 1; 4.95% (IQR 1.83–11.5%), Cluster 2; 7.75% (2.63–14.9%), and Cluster 3; 7.12% (2.54–13.8%) (p = 0.030).

**Table 4:** Comparison of Baseline and RFA Models: Key Biomarkers and CVP associations and regression outcomes by Cluster.

Model	Cluster	Key Biomarkers	CVP Prevalence	Adjusted Odds Ratio, (95% CI)
Initial *sCD14, CRP, LBP, IL-1β, IL-2, MIP-1α, D-dimer, IL-12, IL-6, IL-1RA, TNF-α, IL-10, IL-18, E-selectin, P-selectin, CD40L, vWF, sCD163, MCP-1, ICAM-1, VCAM-1, IFN-γ, I-FABP, TSLP.*	1 (N = 181)	**Low** sCD40L, CD163, CRP, IL18, IL6, and TNF-α	28%	Ref
	2 (N = 183)	**Elevated** sCD40L, IL6, TNF-α, vWF, E-selectin, P-selectin, IL-10	38%	1.17 (0.71, 1.93)
			**Low** IL2, IL12, IL1β	
	3 (N = 44)	**Elevated** IFN-γ, IL1β, IL2, IL12, TNF-α, and MIP1α.	39%	1.30 (0.59, 2.82)
Model 1 *Initial + CXCL9, IL-17, EGF, IL-8, Thrombopoietin, GDF-15*	1 (N = 141)	**Low** TNF-α, CXCL9, IL6, IL1RA, TGF-α, EGF	26%	Ref
	2 (N = 200)	**Elevated** IL-8, IL-10, CRP, TNF-α, GDF-15	35%	1.06 (0.62, 1.82)
			**Low** IL-1β, IL12, MIP-1α2	
	3 (N = 67)	**Elevated** TNF-α, IL6, sCD40L, CXCL9, MCP-1	45%	1.68 (0.85, 3.33)
Model 2 *Initial + CXCL9, EGF, GDF-15, GM-CSF, IFN-α2a, IFN-λ2, IL-5, IL-17, IL-8 Thrombopoietin, TGF-α*	1 (N = 180)	**Low** sCD40L, CXCL9, GDF-15, IFN-λ2, IL-6, IL-17, IL1RA, TGF-α, Thrombopoietin, TNF-α	28%	Ref
	2 (N = 192)	**Elevated** of sCD40L, CXCL9, EGF, GDF-15, IFN-λ2, IL-6, IL-18, MCP-1, TGF-α, and TNF-α,	36%	1.18 (0.72, 1.93)
			**Low** GM-CSF, IFN-α2a, IL-2, and IL-12	
	3 (N = 36)	**Elevated** GM-CSF, IFN-α2a, IFN-γ, IL-1β, IL-2, IL-12, MIP-1α, TSLP, TGF-α, and Thrombopoietin	42%	2.30 (1.05, 5.06)
			**Low** P-Selective	
Model 3 *Initial + IL-17, TGF-α, GDF-15, MDC and IFN- α2a.*	1 (N = 188)	**Low** sCD40L, E-selectin, GDF-15, IL-18, IL-6, IL1RA, MCP-1, Thrombopoietin, TNF-α, vWF	29%	Ref
	2 (N = 181)	**Elevated** P-selectin, TNF-α, and vWF	37%	1.15 (0.71, 1.87)
			**Low** IL-2, IL-12, IFN- α2a.	
	3 (N = 39)	**Elevated** TSLP, IFN-γ, IL-1β, IL-6, IL-12, MIP-1α, TNF-α	38%	1.39 (0.62, 3.06)
			**Low** VCAM	

**Fig 3:**
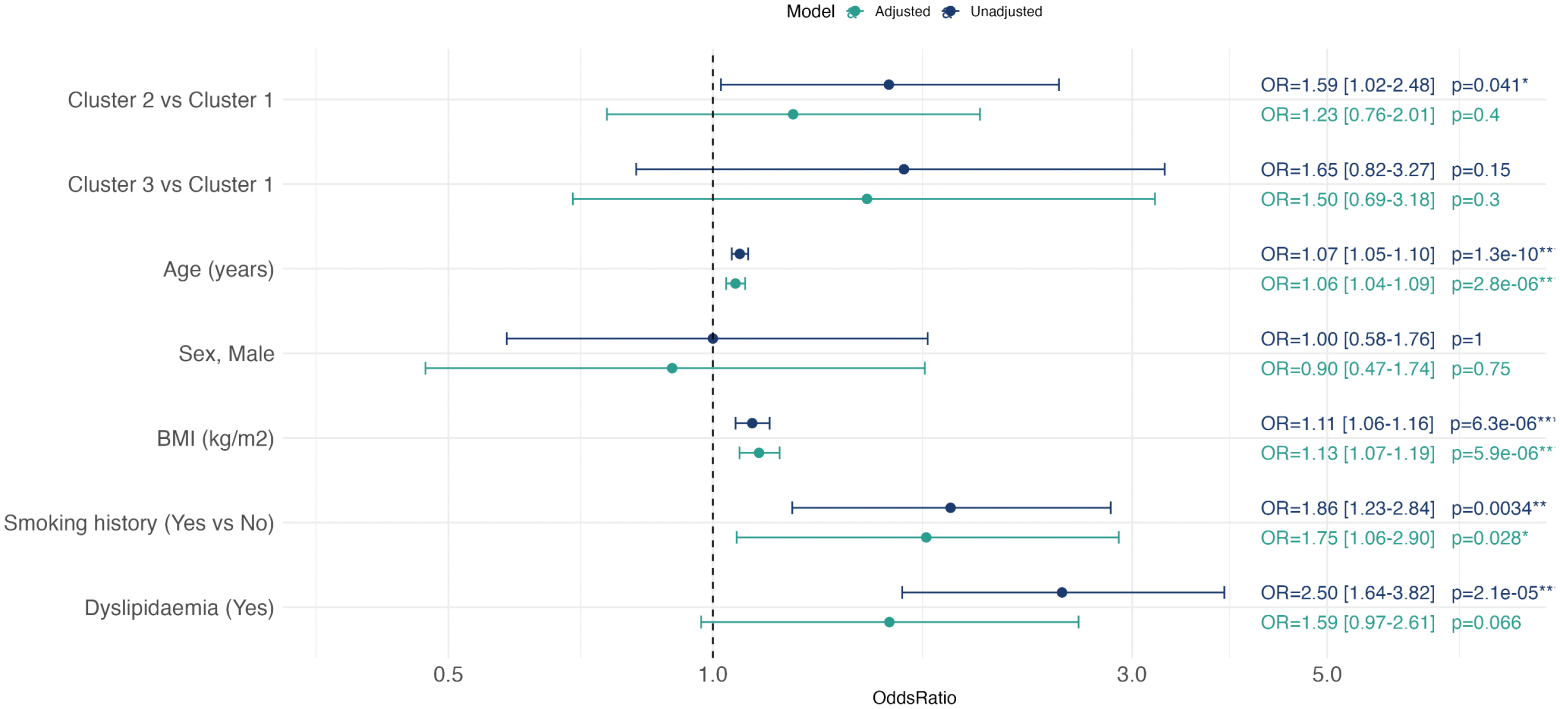
Baseline 24-biomarker model: associations with CVP.

In univariate analyses, older age (OR 1.07 per 1 year increment; 95% CI 1.05–1.10; p < 0.001), higher BMI (OR 1.11 per 1 kg/m^2^ increment; 95% CI 1.06–1.16; p < 0.001), smoking history (current or ex-smoker) (OR 1.86; 95% CI 1.23–2.84; p = 0.003), and dyslipidaemia (OR 2.50; 95% CI 1.64–3.82; p < 0.001) were all associated with greater odds of CVP (Fig 3). Membership in Cluster 2 was also associated with significantly higher CVP odds (OR 1.59; 95% CI 1.02–2.48; p = 0.041) (S1 Table). Although not significant, cluster 3 showed a similar trend toward increased CVP risk (OR 1.65; 95% CI 0.82–3.27; p = 0.15). However, after adjustment, the associations for Cluster 2 (OR 1.23; 95% CI 0.76–2.01, p = 0.55) was attenuated and no longer significant (S2 Table). Univariate analyses determining associations with 10-year ASCVD risk as a continuous outcome yielded broadly similar findings. As with CVP, membership in Cluster 2 was associated with higher ASCVD scores (fold-change 1.40; 95% CI 1.10–1.78; p = 0.005), whereas Cluster 3 was not (fold-change 1.30; 95% CI 0.88–1.91; p = 0.18).

Forest plot showing unadjusted and adjusted logistic regression analyses for inflammatory clusters and clinical covariates in relation to CVP, using cluster 1 as the reference group. Cluster membership was derived from 24 initial inflammatory biomarkers. Adjusted models included age, sex, BMI, smoking history, and dyslipidaemia. Points represent odds ratios with 95% confidence intervals.

### Model 1: Stepwise Addition with Cumulative Evaluation

This model, which sequentially added biomarkers cumulatively to the baseline model, resulted in the selection of 6 additional biomarkers that had the highest impact on model accuracy and multicollinearity; CXCL9, IL-17, EGF, IL-8, Thrombopoietin and GDF-15 (Table 5). Biomarkers identified in the Model 1 analysis showed low to moderate selection frequencies across repeated resamples.

**Table 5:** Biomarker selection criteria scores model 1.

Marker number	Biomarker	VIF Percentage Importance	Accuracy	Kappa	Selection Frequency
Marker27	Thrombopoietin	5.834787	0.876033	0.786671	0.06
Marker30	CXCL9	16.93011	0.892562	0.814066	0.14
Marker32	IL-8	4.237103	0.876033	0.784646	0.16
Marker33	IL-17	4.435918	0.876033	0.787967	0.02
Marker41	GDF-15	5.787582	0.884298	0.797611	0.14
Marker43	EGF	5.310616	0.876033	0.783774	0.32

Candidate biomarkers selected based on their contribution to model performance when added individually to the baseline 24-marker model. VIF percentage importance reflects relative variable contribution, while accuracy and Cohen’s kappa quantify classification performance. Selection frequency (range 0–1) denotes the proportion of repeated resampling iterations in which a biomarker produced a reproducible improvement in both accuracy and kappa relative to the baseline model, serving as a measure of selection stability rather than effect size. Higher values denote greater selection stability; lower values indicate less reproducible performance gains.

**Fig 4:**
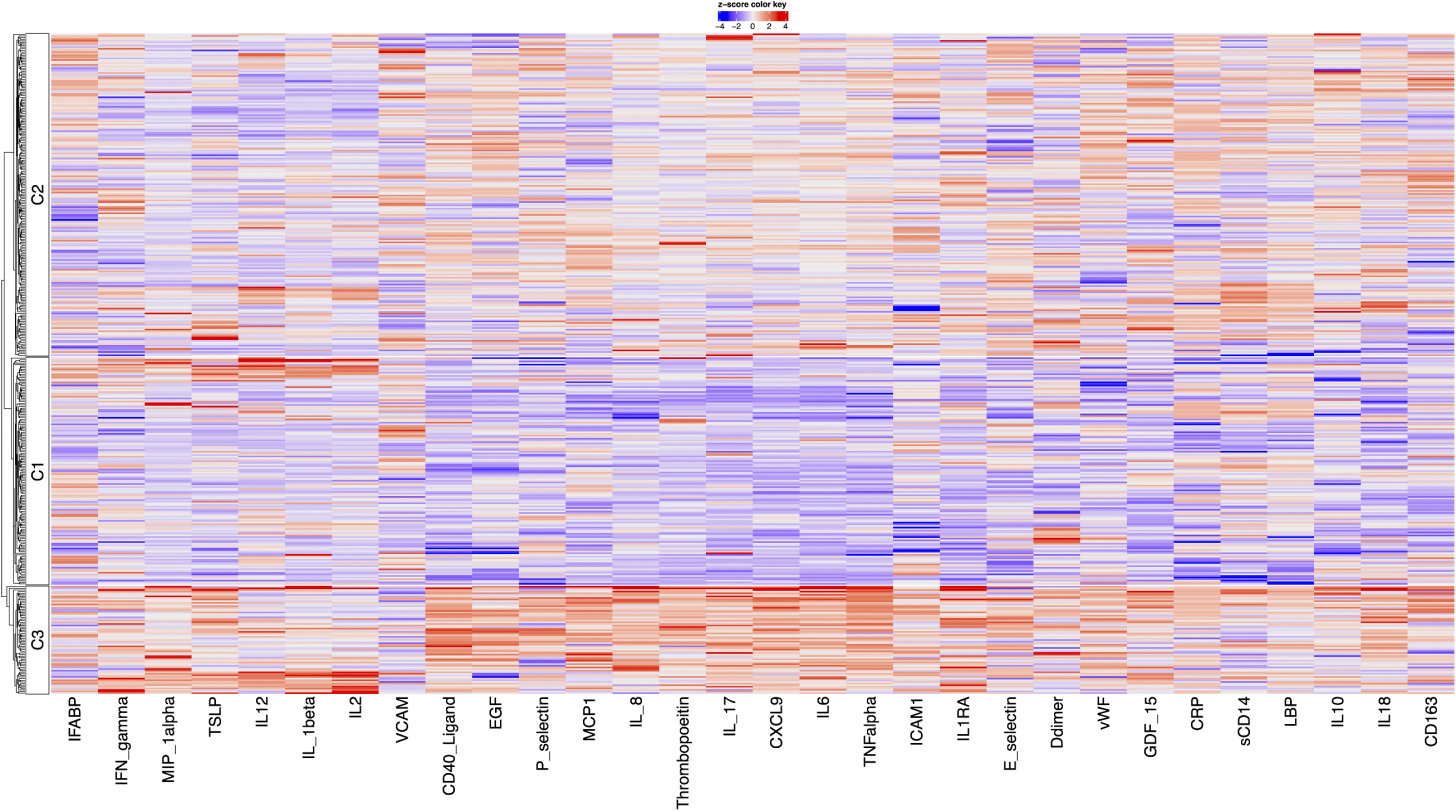
Heatmap showing biomarker contribution to cluster formation in RFA model 1.

Repeat PCA of these 6 markers added to the 24 initial biomarkers yielded 3 clusters with cluster 1 (n = 141, 35%), again displaying low levels of inflammation with suppressed TNF-α, CXCL9, IL6, IL1RA, TGF-α, EGF, Cluster 2 (n = 200, 49%) characterised by elevated IL18, IL10, CRP, TNF-α, GDF-15 and decreased IL-1β, IL12, MIP-1α2 whilst cluster 3 (n = 67, 16.4%) displayed an overall inflamed pattern with high TNF-α, IL1β, IL2, IL-8, CRP, MIP1α (Fig 4). Cluster reproducibility was assessed using 500 bootstrap iterations of PCA and HC. Bootstrap clustering consistently reproduced the same number of clusters as identified in the primary analysis, allowing direct comparison of cluster assignments across resampled datasets. Cluster assignments from each bootstrap were compared to the original solution using the ARI. The distribution of ARI values for model 1indicated moderate cluster stability, with a median Adjusted Rand Index of 0.55 and standard deviation of 0.21, across 500 iterations (S3 Table).

CVP rates in Model 1 varied significantly across clusters, with the highest rate observed in Cluster 3 (45%), compared to Cluster 1 (35%) and Cluster 2 (26%) (p = 0.018). Median 10-year ASCVD risk scores showed a similar pattern, Cluster 1: 4.02% (1.26–10.5%); Cluster 2: 7.58%, (2.97–15.6%); Cluster 3: 8.64% (3.06–14.9%), Kruskal-Wallis p = < 0.0005) (Table 6).

**Table 6:** Median ASCVD 10-year risk and fold-change associations with CVP across models and clusters.

Model	Cluster	ASCVD 10-year risk score, median (IQR)	Fold change, unadjusted (95% CI)
Initial	1	4.95 (1.83–11.5)	Reference
	2	7.75 (2.63–14.9)	1.40 (1.10–1.78), *p* = 0.005
	3	7.12 (2.54–13.8)	1.30 (0.88–1.91), *p* = 0.18
Model 1	1	4.02 (1.26–10.5)	Reference
	2	7.58 (2.97–15.6)	1.65 (1.29–2.12), *p* < 0.001
	3	8.64 (3.06–14.9)	1.74 (1.25–2.44), *p* = 0.0012
Model 2	1	4.9 (1.4–10.8)	Reference
	2	7.7 (2.9–15.5)	1.48 (1.17–1.88), *p* = 0.001
	3	8.4 (2.9–14.3)	1.48 (0.99–2.22), *p* = 0.053
Model 3	1	4.9 (1.8–11.5)	Reference
	2	7.7 (2.9–14.9)	1.41 (1.11–1.79), *p* = 0.005
	3	8.0 (2.7–14.0)	1.36 (0.91–2.04), *p* = 0.13

**Fig 5:**
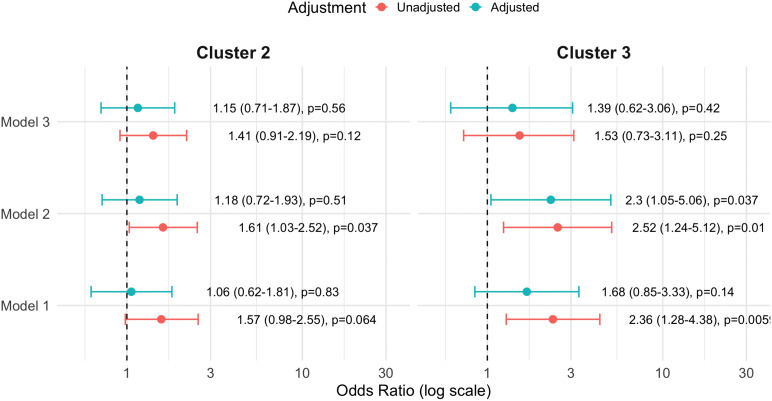
Unadjusted and adjusted odd ratios (95% CI) for CVP in all three RFA models, using cluster 1 as the reference group.

In Model 1, univariate analysis showed that membership in Cluster 2 was not significantly associated (OR 1.57, 95% CI 0.98–2.55) but Cluster 3 membership was associated with higher odds of CVP (OR 2.36, 95% CI 1.28–4.38) (S4 Table). However, after adjustment, Cluster 3 (OR 1.68, 95% CI 0.84–3.32) no longer remained significantly associated with CVP (Fig 5) (S5 Table). A similar pattern was observed when 10-year ASCVD risk was examined, with both clusters 2 and 3 associated with higher ASCVD scores (Cluster 2 fold change 1.65, 95% CI 1.29–2.12, p < 0.001, Cluster 3 fold change 1.74, 95% CI 1.25–2.44, p = 0.0012).

Stratified bootstrap analyses showed that odds ratio estimates were directionally consistent (S6 Table) and robust to sampling variability. When the CVP was stratified into cardiovascular events and hypertension, associations with cardiovascular events were less stable across resamples, whereas associations with hypertension were more consistent, particularly for Cluster 3.

### Model 2: Independent addition without order assumptions

This model, which assessed each marker individually, resulted in 11 biomarkers being added to the baseline model; CXCL9, IL-17, IL-8, Thrombopoietin, GM-CSF, GDF-15, IFN-λ2, IFN-α2a, IL-5, EGF, TGF-α (Table 7). Biomarkers identified in the Model 2 analysis showed low to moderate selection frequencies across repeated resamples.

**Table 7:** Biomarker selection criteria scores model 2.

Biomarker number	Biomarker	VIF Percentage Importance	Accuracy	Kappa	Selection Frequency
Marker27	Thrombopoietin	8.363	0.901	0.829	0.06
Marker30	CXCL9	13.603	0.909	0.843	0.14
Marker32	IL-8	5.984	0.926	0.872	0.16
Marker33	IL-17	5.690	0.876	0.784	0.02
Marker37	IL-5	3.996	0.884	0.795	0.08
Marker41	GDF-15	4.646	0.893	0.812	0.14
Marker42	GM-CSF	4.748	0.909	0.841	0.2
Marker43	EGF	3.873	0.876	0.778	0.32
Marker44	TGF-α	13.160	0.868	0.768	0.2
Marker50	IFN-α 2a	4.008	0.876	0.779	0.1
Marker53	IFN-λ2	3.890	0.901	0.828	0.2

Candidate biomarkers selected based on their contribution to model performance when added individually to the baseline 24-marker model. VIF percentage importance reflects relative variable contribution, while accuracy and Cohen’s kappa quantify classification performance. Selection frequency (range 0–1) denotes the proportion of repeated resampling iterations in which a biomarker produced a reproducible improvement in both accuracy and kappa relative to the baseline model, serving as a measure of selection stability rather than effect size. Higher values denote greater selection stability; lower values indicate less reproducible performance gains.

**Fig 6:**
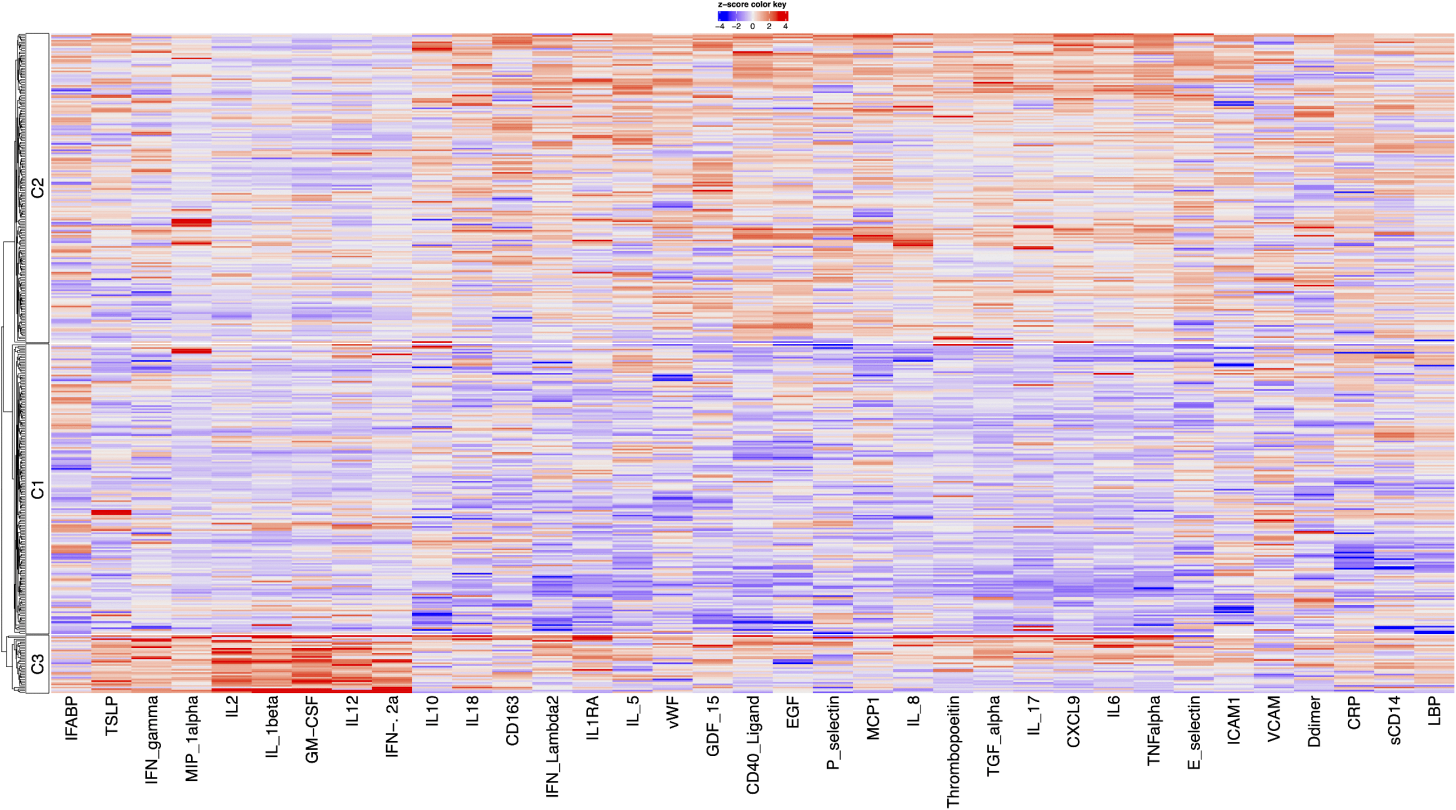
Heatmap showing biomarker contribution to cluster formation in model 2.

Repeat PCA on this expanded biomarker set again identified three distinct clusters (Fig 6). Cluster 1 (n = 180) was characterised by low levels of inflammatory markers, including sCD40L, CXCL9, GDF-15, IFN-λ2, IL-6, IL-17, IL1RA, TGF-α, Thrombopoietin, and TNF-α. Cluster 2 (n = 192) included individuals with elevated levels of sCD40L, CXCL9, EGF, GDF-15, IFN-λ2, IL-6, IL-18, MCP-1, TGF-α, and TNF-α, and low levels of GM-CSF, IFN-α2a, IL-2, and IL-12. Similar to model 1, Cluster 3 (n = 36) was the smallest cluster and exhibited high levels of inflammatory markers such as IL-1β, IL-2 and MIP-1α. However, unlike model 1, this revised cluster 3 also additional markers of innate and adaptive immunity, including elevated GM-CSF, IFN-α2a, IFN-γ, IL-12, TSLP, TGF-α, and Thrombopoietin, with low P-selectin. Bootstrap re-clustering of the expanded biomarker set demonstrated consistent cluster numbers, and good stability of the resulting cluster structure, with a median ARI of 0.74 and standard deviation of 0.16 across 500 bootstrap iterations.

When examining CVP outcomes, Cluster 3 had the highest proportion of CVP (42%), followed by Cluster 2 (36%) and Cluster 1 (28%) (p = 0.14). Median 10-year ASCVD risk scores also differed significantly across clusters, with Cluster 1 at 4.9% (1.4–10.8%), Cluster 2 at 7.7% (2.9–15.5%), and Cluster 3 highest at 8.4% (2.9–14.3%), Kruskal-Wallis p = 0.008).

In univariate analysis, both Cluster 2 (OR 1.61; 95% CI 1.03–2.52; p = 0.037) and Cluster 3 (OR 2.52; 95% CI 1.24–5.12; p = 0.010) were associated with higher odds of CVP compared with the uninflamed Cluster 1 (Fig 5) (S7 Table). After adjustment, only Cluster 3 remained significantly associated with CVP (OR 2.30; 95% CI 1.05–5.06; p = 0.037) (S8 Table). Similarly, for 10-year ASCVD risk, Cluster 2 (fold change 1.48; 95% CI 1.17–1.88; p = 0.001) and Cluster 3 (fold change 1.48; 95% CI 0.99–2.22; p = 0.053) showed higher risk.

Stratified bootstrap analyses demonstrated that Cluster 3 membership was consistently associated with higher odds of the CVP, with a median odds ratio of 2.25 and the odds ratio exceeding unity in 97.5% of bootstrap iterations (S6 Table). When the CVP was stratified, associations with cardiovascular events remained was less stable across resamples, whereas associations with hypertension were more consistent for both clusters.

### Model 3: Bidirectional feature selection

Model 3 applied both forward and backward selection methods to include only biomarkers that improved model performance metrics in both directions. Five biomarkers were ultimately retained: IL-17, TGF-α, GDF-15, MDC and IFN- α2a (Table 8). In repeated resampling analyses, the greedy forward–backward selection procedure showed substantial instability. Across 50 repeated train–test splits, no candidate biomarker (markers 25–55) was selected in more than 10% of runs, and the number of added biomarkers varied between 0 and 3 per iteration.

**Table 8:** Model 3 (Greedy forward - backward) biomarker selection path and resampling stability.

Round	Added Marker	Biomarker	Accuracy	Selection Frequency
**1**	Marker33	IL-17	0.884	0.000
**2**	Marker44	TGF-α	0.901	0.033
**3**	Marker41	GDF-15	0.901	0.000
**4**	Marker29	MDC/CCL22	0.909	0.033
**5**	Marker50	IFN-α 2a	0.917	0.033

Biomarker addition order and accuracy during the greedy forward selection phase of Model 3. Selection frequency represents the proportion of repeated resampling runs in which each biomarker was selected. Model 3 uses a path-based greedy optimisation approach and therefore does not produce marker-level VIF or Kappa metrics, unlike Models 1 and 2.

**Fig 7:**
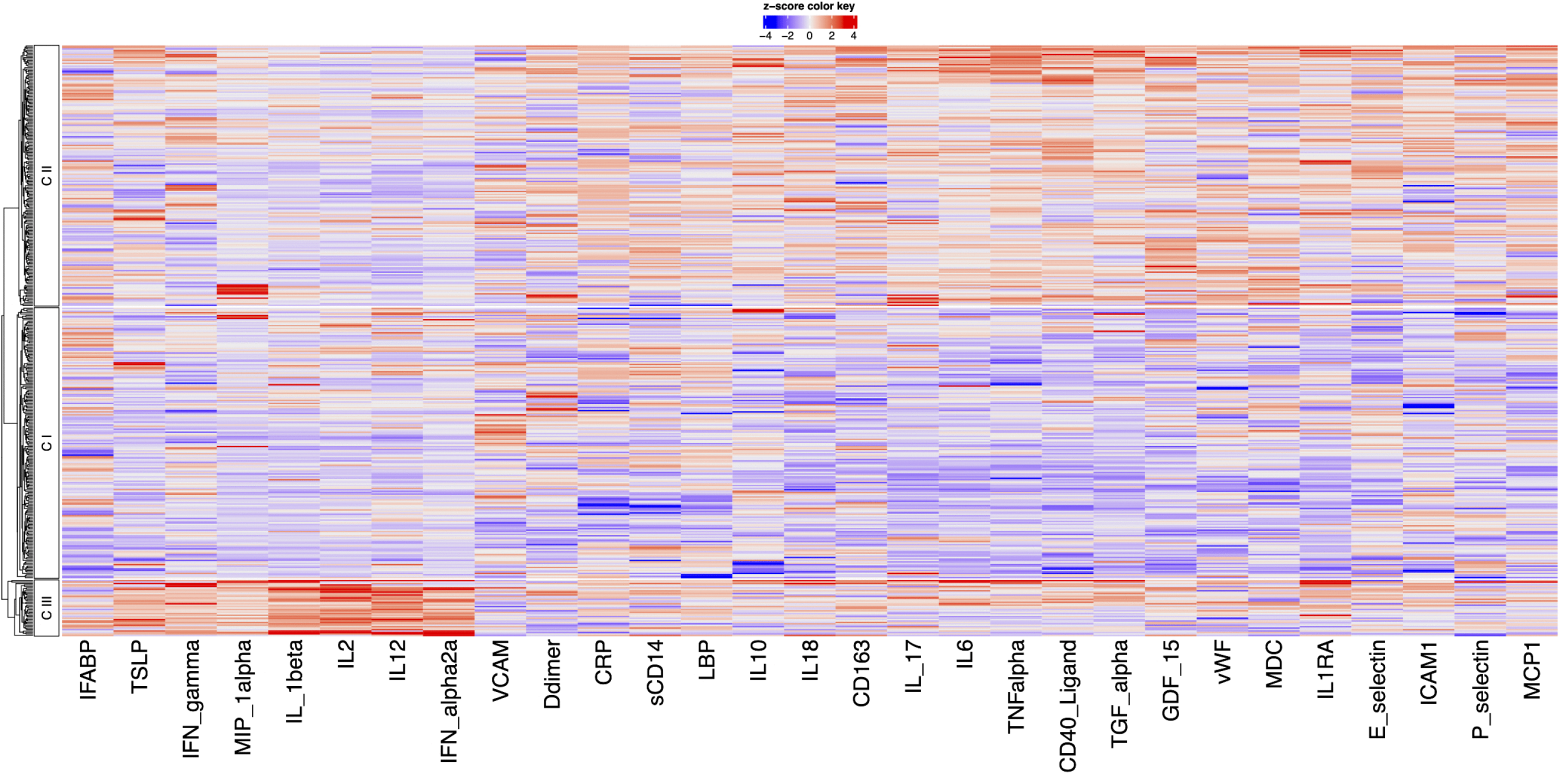
Correlation Heatmap showing biomarker contribution to cluster formation in model 3.

PCA based on these markers once again revealed three clusters (Fig 7). Cluster 1 (n = 186) was characterised by lower inflammatory markers, with reduced levels of sCD40L, E-selectin, CRP, IL-18, IL-6, IL1RA, MCP-1, TNF-α, and vWF. Cluster 2 (n = 183) showed elevated pro-inflammatory and endothelial markers, including P-selectin, TNF-α, and vWF, but low levels of adaptive immune markers like IL-2, IL-12, and IFN- α2a. Cluster 3 (n = 39) exhibited higher levels of immune activation, with high levels of TSLP, IFN-γ, IL-1β, IL-6, IL-12, MIP-1α, and TNF-α, and low expression of the endothelial marker VCAM, reflecting dysregulated adaptive and innate immune responses. Bootstrap re-clustering of the expanded biomarker set demonstrated good stability of the resulting cluster structure, with a median ARI of 0.79 and standard deviation of 0.17 across 500 bootstrap iterations.

Clusters 2 and 3 exhibited the highest CVP prevalence, Cluster 2 (37%), Cluster 3 (38%) compared to Cluster 1 (29%) (p = 0.64). Median ASCVD 10-year risk was likewise highest in Cluster 3 (8.0%, 2.7–14.0) and Cluster 2 (7.7%, 2.9–14.9) compared with Cluster 1 (4.9%, 1.8–11.5) (p = 0.024).

In univariate analysis, neither Cluster 2 (OR 1.41; 95% CI 0.91–2.19; p = 0.12) nor Cluster 3 (OR 1.53; 95% CI 0.73–3.11; p = 0.25) was significantly associated with CVP compared with the uninflamed Cluster 1 (Fig 5) (S9 Table). These were not appreciably altered after adjustment (S10 Table). ASCVD 10-year risk was higher in Cluster 2 (fold change 1.41; 95% CI 1.11–1.79; p = 0.005) but not in Cluster 3 (fold change 1.36; 95% CI 0.91–2.04; p = 0.13) relative to Cluster 1. Bootstrap resampling showed modest, directionally consistent associations between cluster membership and outcomes, but with wide confidence intervals and limited stability across resamples (S6 Table).

### Comparison of biomarker composition across models

**Fig 8:**
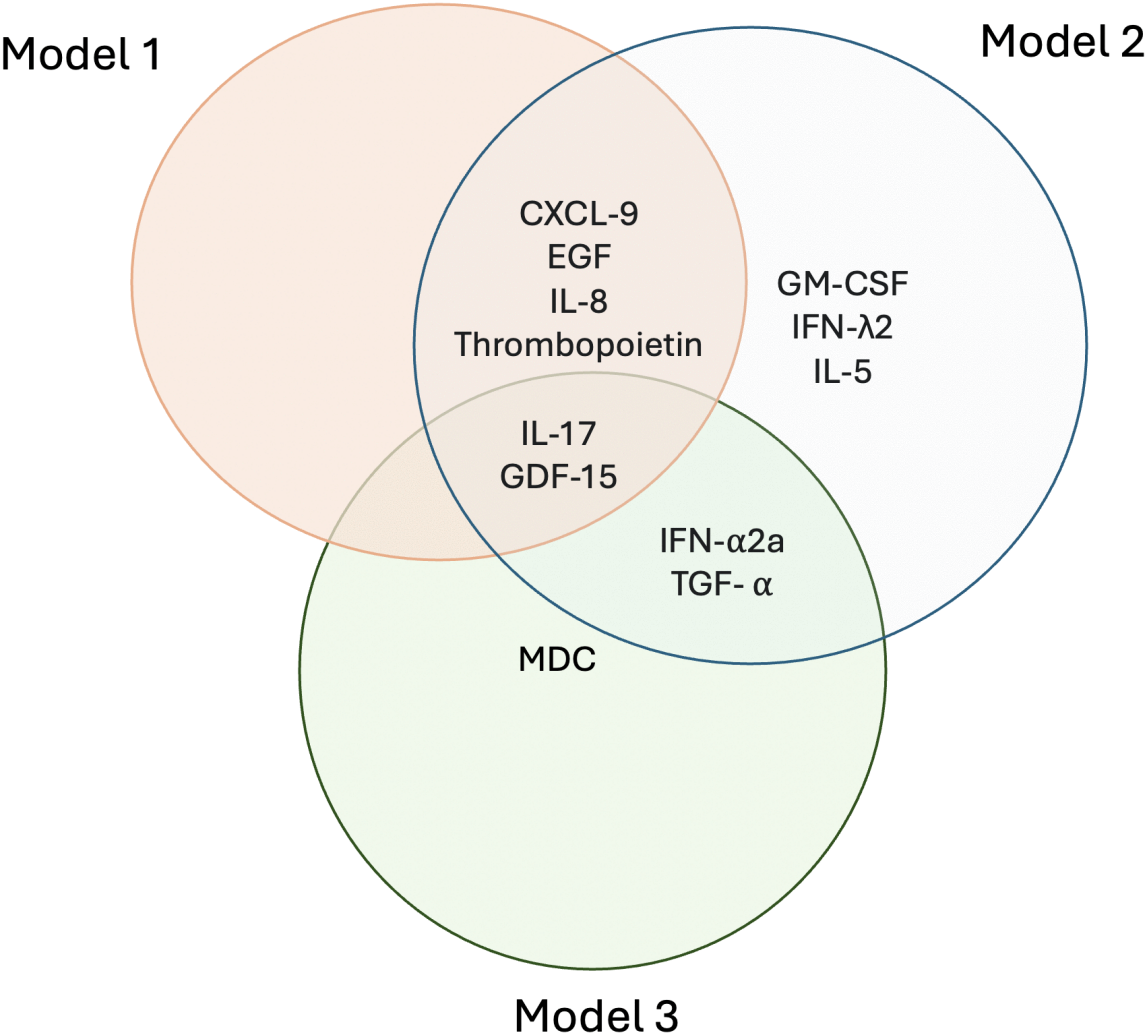
Overlap of Biomarkers Across the Three Predictive Models.

Across models, IL-17 and GDF-15 were consistently selected, underscoring their central roles in inflammatory and vascular processes (Fig 8). Model 1 introduced four additional biomarkers—CXCL9, EGF, IL-8 and Thrombopoietin—broadening coverage of immune activation and growth factor pathways. Model 2 incorporated all of these markers and further expanded the panel to include GM-CSF, IFN-α2a, IFN-λ2, IL-5, and TGF-α, thereby capturing additional interferon and regulatory cytokine pathways. While TGF-α and IFN-α2a were shared with Model 3, the latter retained a more restricted subset, reflecting a narrower focus on immune modulation. Together, these patterns highlight IL-17 and GDF-15 as stable indicators of inflammation related to CVP, with Model 2 offering the most biologically comprehensive framework that was most closely aligned with prevalent CVP.

### Comparison of models and model stability

These three models each evaluated the contribution of additional biomarkers beyond a common initial model and were compared based on biomarker selection reproducibility, cluster stability, and downstream clinical relevance. Overall, selection frequencies indicated low-to-moderate reproducibility of individual biomarkers across resampling iterations, consistent with the presence of correlated inflammatory markers, and stability varied across modelling strategies. Model comparison therefore prioritised unsupervised cluster stability (ARI distributions) and robustness of clinical associations (bootstrap odds ratio distributions), with RF metrics interpreted as secondary indicators of cluster separability. Considering all evaluation criteria, Model 2 demonstrated the strongest overall performance, achieving a balance between robust biomarker selection, stable clustering, and meaningful associations with clinical outcomes (Table 9).

**Table 9:** Comparison of biomarker selection robustness, cluster stability, and bootstrap regression results across models.

Metric	Model 1	Model 2	Model 3
**Biomarker selection strategy**	Stepwise cumulative addition to baseline	Independent single-marker addition to baseline	Greedy forward–backward selection
**Number of additional biomarkers retained**	6	11	5 (path-dependent)
**Selection frequency range (Markers 25–55)**	0.02–0.32	0.02–0.32	0.00–0.03
**Cluster stability (median ARI)**	0.55	0.74	0.79
**Adjusted CVP association (Cluster 3)**	1.68 (0.85-3.33)	2.30 (1.05-5.06)	1.39 (0.62-3.06)
**Composite CVP – median OR (Cluster 3)**	1.71 (0.85–3.56)	2.25 (1.00–5.10)	1.41 (0.64–3.10)
**Composite CVP – OR > 1 (% bootstrap, Cluster 3)**	93.30%	97.50%	80.40%

Biomarker selection stability was assessed using repeated resampling and delta-based improvements in accuracy and Cohen’s kappa relative to the baseline 24-marker model. Cluster stability was quantified using the Adjusted Rand Index (ARI) derived from bootstrap re-clustering. Clinical robustness was evaluated using stratified bootstrap resampling of adjusted logistic regression models for the composite vascular phenotype (CVP) and hypertension. Values are reported as bootstrap median odds ratios (ORs) with 95% percentile-based confidence intervals and the proportion of bootstrap iterations in which ORs exceeded unity

Model 1 identified a limited set of additional biomarkers with low-to-moderate selection frequencies and produced clusters with moderate stability (median ARI 0.55). Although Cluster 3 showed higher associations with the CVP in unadjusted analyses, these associations were attenuated after covariate adjustment and were less stable when cardiovascular outcomes were stratified into clinical events and hypertension.

In contrast, Model 2 identified a broader and more reproducible set of biomarkers that more reliably improved classification beyond the baseline model. Clusters derived under Model 2 exhibited greater stability (median ARI 0.74) and showed greater and more consistent associations with clinical outcomes. In adjusted analyses, Cluster 3 remained significantly associated with CVP, and bootstrap resampling demonstrated a high proportion of iterations in which odds ratios exceeded unity for both CVP and hypertension.

Model 3, which applied a greedy forward–backward selection strategy to iteratively optimise model performance, achieved the highest cluster stability (median ARI 0.79) but showed limited reproducibility in biomarker selection, with most candidate biomarkers selected infrequently across resampling iterations. Correspondingly, associations between cluster membership and clinical outcomes were weaker and less stable, with wide confidence intervals and lower bootstrap consistency, suggesting reduced robustness of downstream inferences despite stable cluster structure.

Although cohort sizes differed substantially, with Dublin contributing the largest number of participants, the distribution of cohorts across clusters was broadly consistent across all clustering models (Table 10). Each model demonstrated similar proportional representation of Amsterdam, Dublin, and London participants within corresponding clusters, indicating that cluster structure was not driven by a single cohort or geographic site. Cohort effects explained a small proportion of variance in the principal component space (S11 Table), and within-cohort regression analyses yielded directionally consistent but imprecise estimates, consistent with limited statistical power in smaller cohorts (S12 Table).

**Table 10:** Distribution of study cohorts across clusters for each clustering model.

Model	Cohort	Cluster 1, n (%)	Cluster 2, n (%)	Cluster 3, n (%)
**Baseline (24 biomarkers)**	Amsterdam	55 (30%)	50 (27%)	7 (16%)
	Dublin	114 (63%)	102 (56%)	28 (64%)
	London	12 (6.6%)	31 (17%)	9 (20%)
**Model 1**	Amsterdam	39 (28%)	65 (33%)	8 (12%)
	Dublin	94 (67%)	104 (52%)	46 (69%)
	London	8 (5.7%)	31 (16%)	13 (19%)
**Model 2**	Amsterdam	57 (32%)	50 (26%)	5 (14%)
	Dublin	114 (63%)	107 (56%)	23 (64%)
	London	9 (5.0%)	35 (18%)	8 (22%)
**Model 3**	Amsterdam	60 (32%)	45 (25%)	7 (18%)
	Dublin	114 (61%)	107 (58%)	23 (59%)
	London	12 (6.5%)	31 (17%)	9 (23%)

Values represent the number and percentage of participants from each cohort within each cluster, stratified by clustering model. Percentages are calculated within cohorts for each model.

## Discussion

In this study, we compared different RFA modelling strategies to enhance biomarker clustering to better associate with CVP in people with HIV. Of the three expanded RFA strategies, an independent single-marker evaluation without cumulative retention approach (Model 2) resulted in the best enhancement of the original clustering to strengthen associations with CVP.

This novel analytical approach provides a structured framework for modelling associations between multiple biomarkers and clinical outcomes, based on an initial choice of markers known to be associated with the outcome, subsequently enriched by additional biomarkers to provide enhanced insights into disease pathogenesis. The result is an enhanced model that represents a meaningful precision medicine approach to help better determine inflammatory patterns associated with important non-communicable diseases.

In our analysis, the initial clustering included a previously characterised panel of 24 biomarkers, chosen for their known association with CAD in people with HIV [5,10]. Compared to previous studies, while this initial clustering successfully stratified individuals into three clusters with distinct inflammatory profiles and moderate differences in CVP prevalence, its discriminatory power in this combined cohort was relatively limited. This may reflect the limited biological scope of the included biomarkers, which, although chosen for their known link to CVP in people with HIV, also capture overlapping aspects of systemic inflammation and immune activation. Building on this initial clustering, our analysis expanded the initial 24 biomarker panel with 31 additional biomarkers encompassing multiple inflammatory, endothelial, and metabolic pathways.

Of the three models explored, Model 2 demonstrated clear advantages over the initial clustering, with the inclusion of 11 biomarkers mapping to pathways relevant to endothelial function, tissue remodelling, and systemic inflammation, aligning well with known CVD mechanisms [23,24]. Compared with Models 1 and 3, Model 2 achieved greater differentiation between clusters, with Cluster 3, characterised by elevated levels of circulating markers of immune regulation (GM-CSF), antiviral activity (IFN-α2a, IFN-γ), systemic inflammation (IL-1β, IL-6), innate immune activation (IL-2, IL-12, TSLP, MIP-1α), and anti-inflammatory activity (IL1RA). Associations between this cluster and CVP persisted after adjustment (adjusted OR: 2.3; 95% CI: 1.04, 5.09), and were supported by stability analyses showing reproducible cluster structure and consistent downstream associations under bootstrap resampling. Although ASCVD scores did not show strong separation between Clusters 2 and 3, the overall pattern supports the biological profile of the clusters and is consistent with the associations observed with the CVP.

Model 1, which resulted in the incorporation of six additional biomarkers, including interferons and growth factors, increased the biological diversity of inflammatory pathways involved but did not improve model performance compared to the baseline model substantially Although this model offered a comprehensive view of immune regulation, the resulting clusters did not show strong differentiation in CVP prevalence, and the association observed for the inflamed cluster in unadjusted analyses did not persist after adjustment. Stability analyses further indicated only moderate reproducibility of the cluster structure and less consistent associations with CVP across resamples, suggesting that the observed effects were sensitive to sampling variability. The ASCVD findings showed a similar effect, with both inflamed clusters exhibiting higher predicted ASCVD risk than the uninflamed Cluster 1, supporting the underlying biological distinctions between clusters, although without strong separation between Cluster 2 and Cluster 3. Although the ASCVD results were favourable and directionally consistent, ASCVD scores were broadly comparable across Models 1 and 2.

Model 3, which employed a greedy backward and forward selection strategy, identified five biomarkers to be added to the initial model. The repeat PCA based on this model again identified an inflamed cluster (Cluster 3) defined by markers such as IL-6, TNF-α, and VCAM-1, all of which align with well-established pathways in inflammation and vascular dysfunction [25–27]. However, performance of this model was less consistent, with lower cluster separation and reduced predictive utility for CVP and despite demonstrating high structural stability of the clustering solution, stability analyses of downstream associations showed limited reproducibility. Additionally, because greedy selection evaluates individual features one step at a time, it may settle on suboptimal sets of biomarkers, missing better combinations that might only emerge when considered in combination, particularly when feature interactions are complex and non-linear. This risk of overfitting and limited generalisability, combined with increased model complexity, may explain why Model 3 was less robust.

Together, these findings highlight how different biomarker integration strategies can yield complementary perspectives on inflammation-driven cardiovascular risk. The consistency of the patterns of cluster across models, with a less inflamed cluster, more inflamed cluster and a third, differentially inflamed cluster 3 all consistently represented, despite varying marker inputs, suggests that a biologically meaningful subset of individuals with distinct altered inflammation can be reproducibly identified.

Interestingly, while the inflamed clusters consistently showed higher observed CVP in unadjusted associations, these effects were mostly attenuated after multivariable adjustment, with the exception of Model 2. Likewise, in analyses of the continuous ASCVD risk score, clusters 2 and 3 both demonstrated higher predicted risk than the uninflamed cluster, but the separation between clusters was limited and similar across models 1 and 2.. This likely reflects the different constructs represented by the two outcomes. The CVP endpoint captures realised disease events, integrating both biological and environmental exposures over time, whereas the ASCVD score estimates predicted risk based on traditional risk factors (age, blood pressure, cholesterol, etc.) that may not fully incorporate the contribution of inflammation. Nevertheless, the complementary nature of these outcomes underscores the added value of biomarker-driven clustering in capturing underlying pathophysiological processes that traditional risk models might overlook.

This analysis demonstrates the utility of an RFA framework for uncovering clinically meaningful groups based on immune and inflammatory biomarker profiles. By iteratively evaluating biomarker contributions to clustering performance, the method enables data-driven identification of informative biomarker panels, improving both interpretability and potential clinical applicability. Compared to traditional clustering approaches, this framework allows for systematic exploration of the importance of each biomarker while maintaining flexibility across diverse datasets and outcomes. The resulting clusters not only reflect underlying biological variation but also show associations with the CVP, highlighting the potential translational relevance of this method. Importantly, the adaptability of this approach enables further modifications as new biomarkers emerge and makes it suitable for application across a range of biomarker-driven disease domains and non-communicable diseases, not just CVD.

Despite the strengths of the RFA framework, this study has several limitations. First, the approach requires access to large, high-quality biomarker datasets to ensure reliable clustering and model stability. Second, the framework is dependent on model-specific performance metrics—such as those derived from RF classifiers—which may introduce bias or limit comparability across different modelling approaches. Additionally, the cross-sectional design limits causal inference and statistical power, while survivor bias and residual confounding cannot be excluded. It is also possible that individuals with established CVD may have modified their behaviour or received interventions that influenced their biomarker profiles, introducing potential reverse causation. Finally, translating this approach to clinical settings poses challenges, as the specialised platforms required to measure these biomarkers are resource-intensive, technically demanding, and not yet integrated into routine diagnostic workflows. Furthermore, the interpretability of machine learning-derived clusters can be complex, potentially limiting clinical uptake without further validation and simplification. To address these limitations, future work should focus on validating the approach in diverse, independent and longitudinal cohorts with incident CVD outcomes to better assess temporal and causal relationships. Additional work should also explore methods to streamline biomarker panels for clinical feasibility, and evaluating ensemble clustering techniques that combine multiple algorithms for greater stability. Integration with broader omics data could also enhance the biological interpretability of clusters, improve predictive power, and potentially reduce reliance on large single-modality biomarker sets.

## Conclusion

In this analysis of a combined, international cohort of people with and without HIV, we identified a RFA framework that can enhance biomarker-derived clustering to predict cardiovascular outcomes. The most robust model, which added biomarkers individually in an unsupervised, data-driven manner, outperformed other models in identifying distinct, outcome-associated clusters. Its adaptive and unbiased design makes it broadly applicable across clinical and biomarker discovery settings, offering an analytical framework for improving host stratification and informing precision medicine for prediction of common comorbidities.

## Supporting information

S1 Table Univariate associations with the composite vascular phenotype for the baseline clustering model. (DOCX)

S2 Table Multivariable associations with the composite vascular phenotype for the baseline clustering model. (DOCX)

S3 Table Bootstrap cluster stability across the three modelling strategies. (DOCX)

S4 Table Univariate associations with the composite vascular phenotype for recursive feature addition Model 1. (DOCX)

S5 Table Multivariable associations with the composite vascular phenotype for recursive feature addition Model 1. (DOCX)

S6 Table Bootstrap sensitivity analysis of cluster–outcome associations across recursive feature addition models. (DOCX)

S7 Table Univariate associations with the composite vascular phenotype for recursive feature addition Model 2. (DOCX)

S8 Table Multivariable associations with the composite vascular phenotype for recursive feature addition Model 2. (DOCX)

S9 Table Univariate associations with the composite vascular phenotype for recursive feature addition Model 3. (DOCX)

S10 Table Multivariable associations with the composite vascular phenotype for recursive feature addition Model 3. (DOCX)

S11 Table Assessment of cohort effects on clustering across recursive feature addition models. (DOCX)

S12 Table Within-cohort adjusted associations between cluster membership and cardiovascular outcomes. (DOCX)

S1 Data Model 1 data archive. Compressed folder containing input biomarker matrices, random forest outputs, cluster assignments, bootstrap resampling results, and summary files used to generate Model 1 results.
(ZIP)

S2 Data Model 2 data archive. Compressed folder containing input data, feature selection outputs, cluster solutions, bootstrap stability metrics, and regression results for recursive feature addition Model 2.
(ZIP)

S3 Data Model 3 data archive. Compressed folder containing greedy forward–backward feature selection outputs, cluster assignments, bootstrap analyses, and supporting files for recursive feature addition Model 3.
(ZIP)

S4 Data R code for recursive feature addition models. Annotated R scripts implementing data preprocessing, imputation, principal component analysis, recursive feature addition strategies, clustering, bootstrap stability analyses, and regression modelling used across all three models.
(ZIP)

S1 Text Membership list for the UPBEAT-CAD, AIID and COBRA cohort working groups. (DOCX)
