# Supplementary material for: Refining biomarker-based clustering of cardiovascular inflammatory phenotypes in HIV using Recursive Feature Addition: A comparative evaluation approach
Source: PLoS Comput Biol. 2026 Apr 27;22(4):e1014209. doi: 10.1371/journal.pcbi.1014209 (PMC13119895; doi:10.1371/journal.pcbi.1014209)
Supplement: S1 Table — (DOCX) [file pcbi.1014209.s001.docx]

# Supplementary Data: Table S1

**Table S 1. Univariate Analysis Results with Odds Ratios for Initial Model**

| Variable | Odds Ratio | P value | CI Low | CI High |
| --- | --- | --- | --- | --- |
| Cluster 2 | 1.58578947 | 0.040978524058336 | 1.021123546 | 2.47574180 |
| Cluster 3 | 1.64962963 | 0.154337292668491 | 0.817850617 | 3.26627679 |
| Age | 1.07288583 | 0.000000000131314 | 1.050798310 | 1.09698493 |
| Smoking | 1.86388385 | 0.003390052780821 | 1.231271128 | 2.83519629 |
| Location, Amsterdam | 1.58507463 | 0.057208966351440 | 0.983252205 | 2.54552045 |
| Location, London | 2.85313433 | 0.000791653135286 | 1.547094860 | 5.29067665 |
| People with HIV | 0.60956938 | 0.043813412167963 | 0.377266258 | 0.99000337 |
| BMI kg/m^2^ | 1.10850176 | 0.000006299960871 | 1.060987269 | 1.16042534 |
| Dyslipidaemia | 2.49639250 | 0.000020953396734 | 1.642289850 | 3.81914901 |
| Elevated Tryglyceride levels | 1.17947960 | 0.085289017655804 | 0.976116295 | 1.42448092 |
| Diabetes History | 2.28453365 | 0.033050673493711 | 1.064348283 | 4.93374453 |

Univariate logistic regression results examining associations between cluster membership and clinical covariates with the composite vascular phenotype outcome. Odds ratios (ORs), 95% confidence intervals (CI), and p-values are reported. Cluster 1 (uninflamed cluster) was used as the reference category
