# Supplementary material for: Refining biomarker-based clustering of cardiovascular inflammatory phenotypes in HIV using Recursive Feature Addition: A comparative evaluation approach
Source: PLoS Comput Biol. 2026 Apr 27;22(4):e1014209. doi: 10.1371/journal.pcbi.1014209 (PMC13119895; doi:10.1371/journal.pcbi.1014209)
Supplement: S2 Table — (DOCX) [file pcbi.1014209.s002.docx]

# Supplementary Data: Table S2

**Table S 2. Multivariate Analysis Results with Odds Ratios for Initial Model**

| Variable | Odds Ratio | P Value | CI Lower | CI Upper |
| --- | --- | --- | --- | --- |
| Cluster 2 | 1.2289071309 | 0.42008 | 0.7443 | 2.0317 |
| Cluster 3 | 1.5649470390 | 0.25961 | 0.7101 | 3.3950 |
| Age | 1.0588082900 | 2.504e-05 | 1.0315 | 1.0880 |
| BMI kg/m^2^ | 1.1330080405 | 5.739e-06 | 1.0751 | 1.1979 |
| Location, Amsterdam | 0.9805913366 | 0.95146 | 0.5192 | 1.8394 |
| Location, London | 1.2863878018 | 0.50596 | 0.6107 | 2.7061 |
| Diabetes History | 1.2646259216 | 0.59787 | 0.5261 | 3.0495 |
| Dyslipidaemia | 1.6238061509 | 0.06517 | 0.9704 | 2.7251 |
| Elevated Triglyceride levels | 0.8803108585 | 0.27736 | 0.6948 | 1.1030 |
| Living with HIV | 0.9161833710 | 0.75859 | 0.5257 | 1.6098 |
| Smoking | 1.7333514797 | 0.03050 | 1.0556 | 2.8657 |

Adjusted logistic regression results assessing associations between cluster membership and the composite vascular phenotype. Models were adjusted for age, body mass index (BMI), smoking status, dyslipidaemia, diabetes history, geographic cohort, and HIV status. Odds ratios (ORs), 95% confidence intervals (CI), and p-values are reported, with Cluster 1 as the reference.
