# Supplementary material for: Refining biomarker-based clustering of cardiovascular inflammatory phenotypes in HIV using Recursive Feature Addition: A comparative evaluation approach
Source: PLoS Comput Biol. 2026 Apr 27;22(4):e1014209. doi: 10.1371/journal.pcbi.1014209 (PMC13119895; doi:10.1371/journal.pcbi.1014209)
Supplement: S3 Table — (DOCX) [file pcbi.1014209.s003.docx]

# Supplementary Data: Table S3

**Table S 3. Bootstrap cluster stability across the three modelling strategies**

| **Model** | **Median ARI** | **Mean ARI** | **SD ARI** | **Min ARI** | **Max ARI** | **Bootstrap Iterations** | **Convergence Failures** |
| --- | --- | --- | --- | --- | --- | --- | --- |
| Model 1 | 0.551 | 0.578 | 0.209 | 0.171 | 0.974 | 500 | <2% |
| Model 2 | 0.736 | 0.694 | 0.169 | 0.273 | 0.984 | 500 | <2% |
| Model 3 | 0.791 | 0.734 | 0.174 | 0.238 | 0.973 | 500 | <2% |

Cluster stability was assessed using bootstrap resampling (500 iterations). In each iteration, participants were sampled with replacement, PCA–HCPC clustering was repeated, and cluster assignments were compared with the original solution using the adjusted Rand index (ARI). ARI values range from 0–1, with higher values indicating greater agreement and stability. Iterations in which clustering failed to converge were excluded (<2%).
