# Supplementary material for: Refining biomarker-based clustering of cardiovascular inflammatory phenotypes in HIV using Recursive Feature Addition: A comparative evaluation approach
Source: PLoS Comput Biol. 2026 Apr 27;22(4):e1014209. doi: 10.1371/journal.pcbi.1014209 (PMC13119895; doi:10.1371/journal.pcbi.1014209)
Supplement: S4 Table — (DOCX) [file pcbi.1014209.s004.docx]

# Supplementary Data: Table S4

**Table S 4. Univariate Analysis Results with Odds Ratios for Model 1**

| Variable | Odds Ratio | P Value | CI Low | CI High |
| --- | --- | --- | --- | --- |
| Cluster 2 | 1.57051282 | 0.063738226379347 | 0.979276214 | 2.54829383 |
| Cluster 3 | 2.36486486 | 0.005883088033336 | 1.282125873 | 4.37884010 |
| Age (years) | 1.07288583 | 0.000000000131314 | 1.050798310 | 1.09698493 |
| Smoking History | 1.86388385 | 0.003390052780821 | 1.231271128 | 2.83519629 |
| Location, Amsterdam | 1.58507463 | 0.057208966351440 | 0.983252205 | 2.54552045 |
| Location, London | 2.85313433 | 0.000791653135286 | 1.547094860 | 5.29067665 |
| Living with HIV | 0.60956938 | 0.043813412167963 | 0.377266258 | 0.99000337 |
| BMI kg/m^2^ | 1.10850176 | 0.000006299960871 | 1.060987269 | 1.16042534 |
| Dyslipidaemia | 2.49639250 | 0.000020953396734 | 1.642289850 | 3.81914901 |
| Elevated Triglyceride levels mmol/L | 1.17947960 | 0.085289017655804 | 0.976116295 | 1.42448092 |
| Diabetes History | 2.28453365 | 0.033050673493711 | 1.064348283 | 4.93374453 |

Univariate logistic regression results examining associations between cluster membership derived from Model 1 and the composite vascular phenotype outcome. Odds ratios (ORs), 95% confidence intervals (CI), and p-values are reported. Cluster 1 was used as the reference category.
