# Supplementary material for: Refining biomarker-based clustering of cardiovascular inflammatory phenotypes in HIV using Recursive Feature Addition: A comparative evaluation approach
Source: PLoS Comput Biol. 2026 Apr 27;22(4):e1014209. doi: 10.1371/journal.pcbi.1014209 (PMC13119895; doi:10.1371/journal.pcbi.1014209)
Supplement: S5 Table — (DOCX) [file pcbi.1014209.s005.docx]

# Supplementary Data: Table S5

**Table S 5. Multivariate Analysis Results with Odds Ratios for Model 1**

| term | Odds Ratio | P Value | CI Lower | CI Upper |
| --- | --- | --- | --- | --- |
| Cluster 2 | 1.043707845 | 0.87587 | 0.6305 | 1.8108 |
| Cluster 3 | 1.680468321 | 0.13292 | 0.8476 | 3.3265 |
| Age | 1.057648526 | 3.705e-05 | 1.0303 | 1.0869 |
| BMI kg/m^2^ | 1.132809990 | 5.743e-06 | 1.0750 | 1.1976 |
| Location, Amsterdam | 1.030822663 | 0.92561 | 0.5427 | 1.9469 |
| Location, London | 1.335188322 | 0.44642 | 0.6328 | 2.8159 |
| Diabetes History | 1.232097911 | 0.63868 | 0.5127 | 2.9644 |
| Dyslipidaemia | 1.664498921 | 0.05418 | 0.9917 | 2.8045 |
| Elevated Triglyceride levels mmol/L | 0.873363380 | 0.24736 | 0.6902 | 1.0942 |
| Living with HiV | 0.937109308 | 0.81911 | 0.5386 | 1.6441 |
| Smoking History | 1.727574164 | 0.03200 | 1.0505 | 2.8599 |

Adjusted logistic regression results for Model 1–derived clusters and the composite vascular phenotype. Models were adjusted for age, BMI, smoking status, dyslipidaemia, diabetes history, geographic cohort, and HIV status. Odds ratios (ORs), 95% confidence intervals (CI), and p-values are reported relative to Cluster 1.
