# Supplementary material for: Refining biomarker-based clustering of cardiovascular inflammatory phenotypes in HIV using Recursive Feature Addition: A comparative evaluation approach
Source: PLoS Comput Biol. 2026 Apr 27;22(4):e1014209. doi: 10.1371/journal.pcbi.1014209 (PMC13119895; doi:10.1371/journal.pcbi.1014209)
Supplement: S6 Table — (DOCX) [file pcbi.1014209.s006.docx]

# Supplementary Data: Table S6

**Table S 6. Bootstrap sensitivity analysis of cluster–outcome associations across recursive feature addition (RFA) models**

| **Model** | **Outcome** | **Cluster** | **Median OR** | **95% CI (Lower–Upper)** | **Proportion OR > 1** |
| --- | --- | --- | --- | --- | --- |
| **Model 1** | Composite Vascular Phenotype | Cluster 2 | 1.03 | 0.57–1.70 | 0.56 |
|  |  | Cluster 3 | 1.71 | 0.85–3.56 | 0.93 |
|  | Cardiovascular events | Cluster 2 | 0.41 | 0.18–1.00 | 0.03 |
|  |  | Cluster 3 | 1.14 | 0.35–3.26 | 0.59 |
|  | Hypertension | Cluster 2 | 1.3 | 0.72–2.47 | 0.8 |
|  |  | Cluster 3 | 1.78 | 0.86–3.56 | 0.94 |
| **Model 2** | Composite Vascular Phenotype | Cluster 2 | 1.24 | 0.71–2.02 | 0.77 |
|  |  | Cluster 3 | 2.25 | 1.00–5.10 | 0.98 |
|  | Cardiovascular events | Cluster 2 | 0.73 | 0.33–1.65 | 0.22 |
|  |  | Cluster 3 | 1.31 | 0.27–4.44 | 0.68 |
|  | Hypertension | Cluster 2 | 1.35 | 0.77–2.34 | 0.85 |
|  |  | Cluster 3 | 2.27 | 1.05–5.18 | 0.98 |
| **Model 3** | Composite Vascular Phenotype | Cluster 2 | 1.15 | 0.67–1.91 | 0.71 |
|  |  | Cluster 3 | 1.41 | 0.64–3.10 | 0.8 |
|  | Cardiovascular events | Cluster 2 | 1.05 | 0.48–2.55 | 0.55 |
|  |  | Cluster 3 | 1.32 | 0.26–4.52 | 0.66 |
|  | Hypertension | Cluster 2 | 1.1 | 0.66–1.84 | 0.64 |
|  |  | Cluster 3 | 1.28 | 0.54–2.78 | 0.72 |

Bootstrap resampling results for adjusted logistic regression models assessing associations between cluster membership and cardiovascular outcomes across three recursive feature addition (RFA) models. Odds ratios (ORs) are reported relative to the uninflamed reference cluster (Cluster 1). Median ORs and percentile-based 95% confidence intervals (CI) were calculated across bootstrap iterations. The proportion of bootstrap samples in which the OR exceeded unity (Proportion OR > 1) provides a measure of directional consistency and robustness of associations.
