# Supplementary material for: Refining biomarker-based clustering of cardiovascular inflammatory phenotypes in HIV using Recursive Feature Addition: A comparative evaluation approach
Source: PLoS Comput Biol. 2026 Apr 27;22(4):e1014209. doi: 10.1371/journal.pcbi.1014209 (PMC13119895; doi:10.1371/journal.pcbi.1014209)
Supplement: S11 Table — (DOCX) [file pcbi.1014209.s011.docx]

# Supplementary Data: Table S11

**Table S 11. Assessment of cohort effects on clustering across RFA models**

| **Model** | **Cluster–cohort association (χ² test)** | **p-value** | **PC1 variance explained (%)** | **Geography effect on PC1 (ANOVA p)** | **Geography effect on PC2 (ANOVA p)** |
| --- | --- | --- | --- | --- | --- |
| Model 1 | χ² = 20.81, df = 4 | 0.00035 | 21.27 | 0.003 | 0.777 |
| Model 2 | χ² = 18.46, df = 4 | 0.001 | 21.38 | <0.001 | 0.665 |
| Model 3 | χ² = 15.10, df = 4 | 0.0045 | 19.3 | <0.001 | 0.863 |

*Evaluation of cohort effects on clustering across recursive feature addition (RFA) models. Cluster–cohort associations were assessed using Pearson’s chi-squared tests. Cohort contributions to biomarker variance were examined by testing the effect of geography on the first two principal components (PC1 and PC2) derived from principal component analysis (PCA) of the biomarker matrices used for clustering. The proportion of variance explained by PC1 is shown for each model.*
