# Supplementary material for: Refining biomarker-based clustering of cardiovascular inflammatory phenotypes in HIV using Recursive Feature Addition: A comparative evaluation approach
Source: PLoS Comput Biol. 2026 Apr 27;22(4):e1014209. doi: 10.1371/journal.pcbi.1014209 (PMC13119895; doi:10.1371/journal.pcbi.1014209)
Supplement: S12 Table — (DOCX) [file pcbi.1014209.s012.docx]

# Supplementary Data: Table S12

**Table S 12. Within-cohort adjusted associations between cluster membership and clinical outcomes across Models 1–3**

| Model | Cluster | Odds Ratio | standard error | P value | CI low | CI high | Geographical location | Outcome |
| --- | --- | --- | --- | --- | --- | --- | --- | --- |
| 1 | Two | 1.620 | 0.422 | 0.253 | 0.718 | 3.794 | Dublin | Hypertension |
|  | Three | 2.691 | 0.498 | 0.047 | 1.017 | 7.274 | Dublin | Hypertension |
|  | Two | 0.329 | 0.610 | 0.069 | 0.092 | 1.053 | Dublin | CVD events |
|  | Three | 1.233 | 0.585 | 0.720 | 0.380 | 3.874 | Dublin | CVD events |
|  | Two | 1.370 | 0.463 | 0.497 | 0.557 | 3.464 | Amsterdam | Hypertension |
|  | Three | 0.649 | 0.936 | 0.645 | 0.079 | 3.638 | Amsterdam | Hypertension |
|  | Two | 0.854 | 1.006 | 0.875 | 0.119 | 7.670 | Amsterdam | CVD events |
|  | Three | 1.47E-08 | 5727.057 | 0.997 | NA | 1.35E+209 | Amsterdam | CVD events |
|  | Two | 0.201 | 1.047 | 0.125 | 0.021 | 1.414 | London | Hypertension |
|  | Three | 0.750 | 1.185 | 0.808 | 0.064 | 7.612 | London | Hypertension |
|  | Two | 0.391 | 1.418 | 0.507 | 0.023 | 10.421 | London | CVD events |
|  | Three | 0.426 | 1.750 | 0.626 | 0.009 | 16.647 | London | CVD events |
| 2 | Two | 2.311 | 0.407 | 0.040 | 1.057 | 5.277 | Dublin | Hypertension |
|  | Three | 5.186 | 0.563 | 0.003 | 1.717 | 15.895 | Dublin | Hypertension |
|  | Two | 0.685 | 0.530 | 0.475 | 0.237 | 1.948 | Dublin | CVD events |
|  | Three | 1.592 | 0.711 | 0.513 | 0.359 | 6.158 | Dublin | CVD events |
|  | Two | 0.701 | 0.442 | 0.420 | 0.290 | 1.657 | Amsterdam | Hypertension |
|  | Three | 0.419 | 1.163 | 0.455 | 0.020 | 3.062 | Amsterdam | Hypertension |
|  | Two | 0.669 | 0.964 | 0.677 | 0.083 | 4.501 | Amsterdam | CVD events |
|  | Three | 3.95E-08 | 6413.770 | 0.998 | NA | 3.81E+258 | Amsterdam | CVD events |
|  | Two | 0.911 | 0.916 | 0.919 | 0.143 | 5.651 | London | Hypertension |
|  | Three | 1.517 | 1.184 | 0.725 | 0.146 | 16.919 | London | Hypertension |
|  | Two | 0.632 | 1.405 | 0.744 | 0.043 | 17.312 | London | CVD events |
|  | Three | 0.281 | 2.131 | 0.551 | 0.001 | 15.274 | London | CVD events |
| 3 | Two | 1.291 | 0.378 | 0.500 | 0.615 | 2.734 | Dublin | Hypertension |
|  | Three | 2.598 | 0.582 | 0.101 | 0.803 | 8.050 | Dublin | Hypertension |
|  | Two | 1.203 | 0.513 | 0.718 | 0.441 | 3.390 | Dublin | CVD events |
|  | Three | 2.086 | 0.780 | 0.346 | 0.391 | 9.077 | Dublin | CVD events |
|  | Two | 0.700 | 0.449 | 0.427 | 0.285 | 1.674 | Amsterdam | Hypertension |
|  | Three | 4.98E-08 | 1466.533 | 0.991 | NA | 3.38E+49 | Amsterdam | Hypertension |
|  | Two | 1.045 | 0.900 | 0.961 | 0.163 | 6.502 | Amsterdam | CVD events |
|  | Three | 4.41E-08 | 6045.786 | 0.998 | NA | 2.32E+236 | Amsterdam | CVD events |
|  | Two | 2.086 | 0.841 | 0.382 | 0.410 | 11.846 | London | Hypertension |
|  | Three | 3.005 | 1.129 | 0.330 | 0.345 | 31.991 | London | Hypertension |
|  | Two | 0.315 | 1.363 | 0.397 | 0.019 | 5.662 | London | CVD events |
|  | Three | 0.214 | 2.006 | 0.442 | 0.001 | 6.162 | London | CVD events |

*Cohort-stratified adjusted logistic regression results examining associations between cluster membership and hypertension or adjudicated cardiovascular disease events across Models 1–3. Odds ratios are reported relative to Cluster 1 and were adjusted for age, body mass index, smoking status, sex (where applicable), and dyslipidaemia. Analyses were performed separately within each geographic cohort to assess the robustness of cluster–outcome associations to cohort-specific effects*
