# Supplementary material for: Refining biomarker-based clustering of cardiovascular inflammatory phenotypes in HIV using Recursive Feature Addition: A comparative evaluation approach
Source: PLoS Comput Biol. 2026 Apr 27;22(4):e1014209. doi: 10.1371/journal.pcbi.1014209 (PMC13119895; doi:10.1371/journal.pcbi.1014209)
Supplement: S1 Text — (DOCX) [file pcbi.1014209.s017.docx]

# Supplementary Data: Text S1

**HIV UPBEAT Study Group.** Centre for Experimental Pathogen Host Research, University College Dublin, Ireland: P. McGettrick, E. Alvarez Barco, W. Tinago, A. Garcia-Leon, A. McDermott, T. McGinty, A. G. Cotter, A. Macken, P. W. G. Mallon. Mater Misericordiae University Hospital: E. Kavanagh, G. McCarthy, G. Sheehan, J. Lambert. Institute for Public Health, Washington University School of Medicine, St Louis, Missouri: W. Powderly. Department of Medicine, Cambridge Biomedical Campus, Cambridge, UK: J. Compston. Institute for Global Health, University College London, UK: C. Sabin.

**All-Ireland Infectious Diseases Cohort Study Group**. Mater Misericordiae University Hospital: A. Cotter, E. Muldoon, G. Sheehan, T. McGinty, J. S. Lambert, S. Green, K. Leamy. St Vincent's University Hospital: G. Kenny, K. McCann, R. McCann, P. Smolovyk, F, Rigonat, C. O’Broin, S. Waqas, S. Savinelli, E. Feeney, P. W. G. Mallon. Centre for Experimental Pathogen Host Research: A. Garcia Leon, S. Miles, D. Alalwan, R. Negi. Beaumont Hospital: E. de Barra, S. McConkey, K. Hurley, I. Sulaiman. University College Cork: M. Horgan, C. Sadlier, J. Eustace. University College Dublin: C. Kelly, T. Bracken. Sligo University Hospital: B. Whelan. Our Lady of Lourdes Hospital: J. Low. Wexford General Hospital: O. Yousif. University Hospital Galway: B. McNicholas. St Luke's Hospital Kilkenny: G. Courtney. Children's Health Ireland: P. Gavin.

**Co-morbidities in relation to HIV/AIDS Study Group.** D. De Francesco, F.W. Wit, J.H. Cole, N.A. Kootstra, A. Winston, C.A. Sabin, J. Underwood, R.A. van Zoest, J. Schouten, K.W. Kooij, M. Prins, G. Guaraldi, M.W.A. Caan, D. Burger, C. Franceschi, C. Libert, A. Bürkle, P. Reiss, M.F. Schim van der Loeff, B. Schmand, G.J. Geurtsen, D.J. Sharp, C. Majoie, J. Villaudy, B. Berkhout, M. Gisslén, A. Pasternak, M. van der Valk, P.H. Bisschop, P. Portegies, S. Zaheri, W. Zikkenheiner, F.R. Janssen, N. Doyle, E. Verheij, S.O. Verboeket, B.C. Elsenga, M.M.J. Hillebregt, Y.M.C. Ruijs, D.P. Benschop, L. Tembo, L. McDonald, M. Stott, K. Legg, A. Lovell, O. Erlwein, C. Kingsley, P. Norsworthy, S. Mullaney, T. Kruijer, L. del Grande, V. Olthof, G.R. Visser, L. May, F. Verbraak, N. Demirkaya, I. Visser, C.B.L.M. Majoie, T. Su, R. Leech, J. Huguet, E. Frankin, A. van der Kuyl, K. Weijer, E. Siteur-Van Rijnstra, M. de Graaff-Teulen, A.M. Harskamp-Holwerda, I. Maurer, M.M. Mangas Ruiz, A.F. Girigorie, B. Boeser-Nunnink, T. Booiman, A. Kalsbeek, P.H.L.T. Bisschop, J. Hoeijmakers, J. Pothof, S. Dewaele, P. Garagnani, C. Pirazzini, M. Capri, F. Dall’Olio, M. Chiricolo, S. Salvioli, D. Fuchs, H. Zetterberg, D. Weber, T. Grune, E.H.J.M. Jansen, T. Sindlinger, and S. Oehlke.
